# Neural stimulation and modulation with sub-cellular precision by optomechanical bio-dart

**DOI:** 10.1038/s41377-024-01617-9

**Published:** 2024-09-19

**Authors:** Guoshuai Zhu, Jianyun Xiong, Xing Li, Ziyi He, Shuhan Zhong, Junlin Chen, Yang Shi, Ting Pan, Li Zhang, Baojun Li, Hongbao Xin

**Affiliations:** 1 Guangdong Provincial Key Laboratory of Nanophotonic Manipulation, Institute of Nanophotonics, College of Physics and Optoelectronic Engineering, Jinan University, Guangzhou, 511443 China; 2 Key Laboratory of CNS Regeneration (Ministry of Education), Guangdong-Hong Kong-Macau Institute of CNS Regeneration, Jinan University, Guangzhou, 510632 China

**Keywords:** Optical manipulation and tweezers, Biophotonics, Biomedical materials

## Abstract

Neural stimulation and modulation at high spatial resolution are crucial for mediating neuronal signaling and plasticity, aiding in a better understanding of neuronal dysfunction and neurodegenerative diseases. However, developing a biocompatible and precisely controllable technique for accurate and effective stimulation and modulation of neurons at the subcellular level is highly challenging. Here, we report an optomechanical method for neural stimulation and modulation with subcellular precision using optically controlled bio-darts. The bio-dart is obtained from the tip of sunflower pollen grain and can generate transient pressure on the cell membrane with submicrometer spatial resolution when propelled by optical scattering force controlled with an optical fiber probe, which results in precision neural stimulation via precisely activation of membrane mechanosensitive ion channel. Importantly, controllable modulation of a single neuronal cell, even down to subcellular neuronal structures such as dendrites, axons, and soma, can be achieved. This bio-dart can also serve as a drug delivery tool for multifunctional neural stimulation and modulation. Remarkably, our optomechanical bio-darts can also be used for in vivo neural stimulation in larval zebrafish. This strategy provides a novel approach for neural stimulation and modulation with sub-cellular precision, paving the way for high-precision neuronal plasticity and neuromodulation.

## Abstract (web summary)

We report a non-genetic optical neural stimulation and modulation strategy with sub-cellular precision based on optomechanical bio-dart, which also enables in vivo neural stimulation.

## Graphical abstract

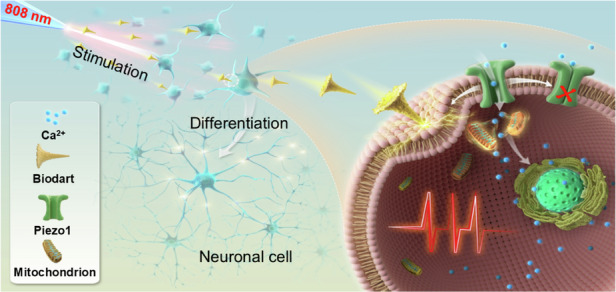

## Introduction

Neurodegenerative diseases (NDDs) are a group of neurological disorders characterized by the breakdown of neural network function and the loss of neurons^1–3^. NDDs, such as Parkinson’s disease, Alzheimer’s disease, and amyotrophic lateral sclerosis, greatly harm the health of millions of people worldwide. These diseases are usually caused by abnormal functional states of neuronal cells or subcellular structures, such as pathological protein aggregation, synaptic dysfunction, neuronal cell death, and cellular cytoskeletal abnormalities^4^. Controllable stimulation and modulation of neurons with high precision will help better understanding of these abnormal functional states of neurons. Neuromodulation techniques, using external stimulation or intervention to modify and to regulate neural activity, have been emerged as a promising method in neural modulation and the treatment of NDDs^5–7^. Electrical stimulation is one of the most important techniques in neural modulation^8^. However, the spatial resolution of electrical stimulation is of low precision and cannot be targeted to individual neurons^9^. In addition, it has no cell-specificity in neural regulation. Transcranial magnetic stimulation, as the most prescribed neuromodulation method, has been used for treating neurological and psychiatric disorders, such as Parkinson’s Disease, depression, and epilepsy^10^. However, magnetic stimulation requires a high-intensity magnetic field with low spatiotemporal resolution, which limits its non-invasive control over the neuronal system^11^. Ultrasound neuromodulation has unique potential to non-invasively control neural activity in deep brain regions without the need for chemical or genetic modifications^12,13^. However, this method is limited by the low spatial resolution^14^. Therefore, there is an urgent need for the development of neural modulation techniques with high bio-compatibility and high spatial resolution.

To increase the stimulation precision, different methods have been emerged. Among them, optical modulation technique has emerged as a promising strategy for neural stimulation due to its inherent advantages such as biological safety^15–17^, ease of operation^18,19^ and high spatiotemporal resolution^20–22^. Optogenetics is currently the most popular optical neural modulation technique, in which cells are genetically introduced with light sensitive proteins to achieve optical modulation of neural activities with excellent spatial and temporal precision^23,24^. However, the complex genetic modifications and unstable gene expression of modified genes pose significant challenges to the practical applications^25,26^. Direct irradiation with infrared light pulses in non-inheritable genetic manner can also be used to activate excitable cells or tissues in the illumination spot^14,27,28^. However, the conversion of electromagnetic waves into thermal energy may cause cellular damage^29,30^. In addition to optical modulation, optoacoustic stimulation has also been used for neural modulation. Using an optoacoustic converter based on an optical fiber with a pulsed light, neural stimulation at a micrometer spatial resolution on a single-cell level has been achieved by exploiting the optoacoustic effect with light absorbing-induced ultrasound wave^6,31,32^. Nevertheless, such resolution is still insufficient for targeting the sub-cellular structures of neurons. New strategies that can be used for neural stimulation and modulation with subcellular precision are in great demand to help better understanding of neural function, growth, signaling and plasticity.

**Fig. 1:**
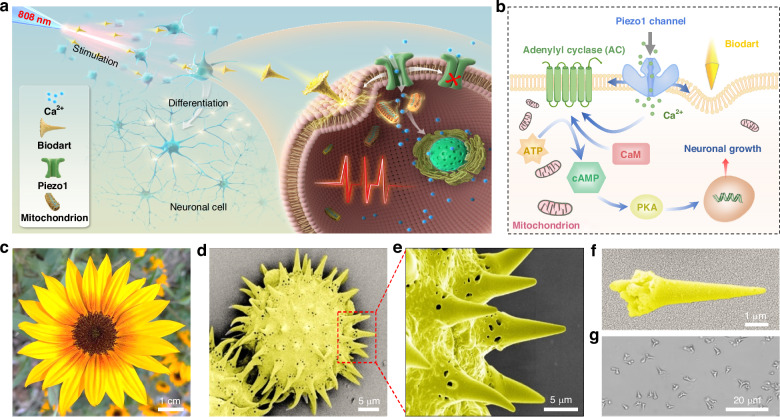
**Precision neural stimulation and modulation with optomechanical bio-dart**. **a** Schematic illustration of precise neural stimulation using optomechnical bio-dart. An 808 nm laser beam is launched into a SMOFP for bio-dart shooting and targeting toward a neural cell for neural stimulation and modulation. **b** Schematic illustration of activation of Piezo1 channel and intracellular pathways by optomechanical dart. **c** Photograph of a natural sunflower. SEM image showing (**d**) a sunflower pollen grain (SPG), (**e**) the spiky structures of SPG, and (**f**) a fabricated bio-dart. (**g**) Microscopic image of darts

In this work, we report a non-genetic neural stimulation and modulation method with sub-cellular precision based on an optomechanical nanotransducer, i.e., an optically controlled bio-dart. The bio-dart, obtained from the tip of sunflower pollen grains, can be targeted shot toward a neural cell by optical scattering force at high speed, enabling neural stimulation and modulation at sub-cellular precision (Fig. 1a). This high-precision neural stimulation and modulation is resulted from the activation of mechanosensitive ion channels (Piezo1 channels) at cell membranes of subcellular structures (such as dendrite, soma, and axon) by the bio-dart induced transient pressure, and subsequent influx of calcium ions and activation of molecular pathway (Fig. 1b). This method can also be translated to in vivo neural stimulation. This strategy serves as a new approach for neural stimulation and modulation at sub-cellular precision, and holds great promises for better understanding of neuronal function/disfunction, growth, signaling, and plasticity with high spatiotemporal resolution.

## Results

### Controllable shooting of bio-dart for subcellular targeting

Pollen microparticles, abundant in nature, represent a highly promising naturally available material with high biocompatibility^33^. In our work, sunflower pollen grains (SPGs) were selected to fabricate the darts (Fig. 1c). The SPGs exhibit a sea urchin-like shape with spiky tip structures (Fig. 1d, e), making SPGs an excellent candidate for exerting a superior mechanical effect on cells, enabling various applications such as targeted drug delivery^34^ and immunity activation^35^ by selectively piercing the cell membrane. This feature inspired us to create an optomechanical nanotransducer based on natural bio-darts using the tips of SPGs. The bio-darts were obtained from the spiky tip of the SPGs, with a conical shape (Fig. 1f, g). Details of the fabrication can be found in the Materials and Methods section and Supplementary Fig. S1 in the Supporting Information. The fabricated darts have an average length of 7.5 μm (Supplementary Fig. S1d), with the tip and base dimensions of approximately 170 nm and 3.1 μm, respectively. The nanoscale conical shape of SPGs tips makes them excellent candidates for bio-darts that can be propelled toward targeted subcellular structures of neural cells.

**Fig. 2:**
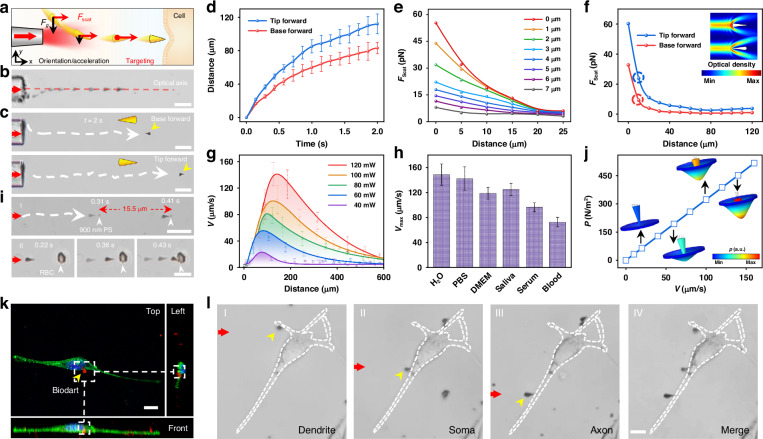
**Optical shooting and mechanical pressure generation of bio-dart**. **a** Schematic illustration of optical shooting of bio-dart toward cell membrane with pressure generation. The shooting process includes the orientation/acceleration movement of the bio-dart aside the central axis of SMOFP, and the targeting movement along the central axis. **b** Microscopic image showing the trajectory of bio-dart with orientation/acceleration and targeting movement. The red arrows indicate the 808 nm laser, and the straight indicates the central axis of SMOFP. **c** Trajectory of bio-dart movement within 2 s with orientation of (I) tip forward and (II) base forward. The curve indicates the moving trajectory of the dart. **d** Moving distance as a function of time for bio-dart with different initial orientation. **e** Calculated optical scattering force (*F*_scat_) as a function of distance from the central axis of the fiber tip. **f** Calculated optical scattering force (*F*_scat_) as a function of distance from the fiber tip, inset shows the simulated optical intensity fields of darts with two different orientations. **g** Velocity of dart as a function of distance at different powers. **h** Measured shooting velocity of the dart in different media. **i** Microscopic images showing bio-dart shooting toward (I) a 900-nm PS particle and (II) a red blood cell. **j** Calculated pressure on cell membrane as a function of dart velocity, inset shows the simulated pressure fields on cell membrane. **k** Different views of confocal laser scanning microscopic image showing neural cell with dart hitting. Green: F-actin, blue: cell nucleus, red: dart. **l** Microscopic images showing precision localization of bio-darts on different sub-cellular structures of (I) dendrite, (II) soma and (III) axon of a neural cell. Panel IV shows the merged figure. The red and yellow arrows indicate 808 nm laser and dart, respectively. Scale bars: 10 µm

For the controllable shooting of the bio-dart, optical gradient force and optical scattering force generated by a laser beam (wavelength: 808 nm) output from a flat-end tapered single-mode optical fiber probe (SMOFP, tip size: 15 μm, details of the fabrication see in Supplementary Fig. S2 and Materials and methods section in the Supporting Information) were applied on the bio-dart^36^. The SMOFP is controlled by a six-axis micromanipulator with a manipulation resolution of 100 nm. Details of the manipulation can be seen in Supplementary Fig. S3 and Materials and methods section in the Supporting Information. This manipulation resolution enables the precision control of the SMOFP, and thus the dart can be precisely controlled to shoot toward the target region of a neuronal cell by optical scattering force along the optical axis of the fiber probe. With the laser beam launched into the SMOFP, as shown in Fig. 2a, the bio-dart aside the central axis of the SMOFP can be trapped toward the central axis by the optical gradient force (*F*_g_) perpendicular to the central axis. Meanwhile, the optical scattering force (*F*_scat_) can cause the bio-darts to move forward along the central axis direction. It should be noted that since the light intensity and scattering force exponentially decreased away from the central axis (Fig. 2e and Supplementary Fig. S4b), the optical scattering force is increased as the dart is trapped toward the central axis (Fig. 2f). This increased scattering force can increase the velocity of the dart. Therefore, when the dart is trapped near the central axis, the dart is also accelerated. This is different from the traditional dart shooting, where the velocity of the dart gradually decreases once it is shot out. This is also the reason why we use a flat-end SMOFP instead of a tapered-end SMOFP for dart shooting. During the process of dart trapping toward the optical axis, the dart is gradually oriented with the long axis along the central axis of the SMOFP. Once trapped on the central axis, the dart can be further moved toward the target region along the central axis by optical scattering force. Once hitting the target region of a neural cell, the nano-tip can generate transient pressure on the cell membrane with a sub-micrometer spatial resolution (Fig. 2a). In this case, the bio-dart is turned into an optomechanical nanotransducer for neural stimulation and modulation. During this process, darts with either base or tip forward can be shot out. However, the size of the tip is smaller than that of the base, therefore, the targeting accuracy for dart with tip forward is larger than that with base forward. Figure 2b shows the process of trapping and orienting the dart toward the central axis. Details of the trapping and orientation can be seen in Supplementary Video S1. Figure 2c shows the trajectories of dart with both the base forward and tip forward (laser power: 120 mW). However, the velocity of the dart with tip facing forward is higher than that with the base facing forward (Fig. 2d). With a moving time of 2 s, the shooting distance of the darts with the tip facing forward was about 112 μm, while that with base facing forward was about 83.2 μm. This is primarily due to the larger scattering force exerted on the dart with the tip facing forward, as shown in Fig. 2f from numerical simulation (Details of the simulation is shown in Supplementary Fig. S5). This larger velocity can result in a larger pressure on the cell membrane. In addition, the smaller area of the tip is also more conducive to generating pressure than that of the larger base. Therefore, in our work, we intentionally chose the dart with tip facing toward the neuron cell for shooting and further for precision neural stimulation and modulation.

During the shooting process, the velocity of the dart gradually increased until reaching a maximum, and then gradually decreased (Fig. 2g). The shooting velocity was dependent on the laser power (Fig. 2g and Supplementary Fig. S6), with the maximal velocity (*V*_max_) of the darts increased from 26 to 148 μm s^−1^, reaching distance (*D*_Vmax_) of 70 to 139 μm, as the optical power increased from 40 to 120 mW. For a power of 120 mW, the value of *V*_max_ (148 μm s^−1^) was about 19.7 body length per second. This velocity is larger than most other micromotors controlled with magnetic fields^37^ and chemical reaction^38^. For further biomedical applications, the motion capability of the darts in different biological media is of significant importance^39,40^. As shown in Supplementary Fig. S7 (Supplementary Video S2), the darts can also be shot out and move in different biological culture media such as water, Phosphate-buffered saline (PBS), cell culture medium (Dulbecco’s Modified Eagle Medium, DMEM), saliva, human serum, and blood, but with different velocities. As shown in Fig. 2h, with the increase in a medium viscosity, the movement speed of darts was decreased, with average maximum speeds of 148, 142, 118, 125, 96, and 73 μm s^−1^ in water, PBS, DMEM, saliva, human serum, and blood, respectively. This controllable shooting capability in various biological media lays the foundation for further biomedical applications. For further applications of neural stimulation and modulation, the bio-compatibility of the darts is very important. We performed additional experiments to show the bio-compatibility of the darts by culturing neural cells (HT22) with the darts. Experimental results show that the darts exhibit good sterility and high bio-compatibility with negligible toxicity, and the cell viability was >85% after 5 days co-culturing with darts (Supplementary Fig. S8), which is only a slight decrease compared with neurons cultured in a normal condition without darts. The proliferation of neurons was also not affected after co-culture with darts (Supplementary Fig. S8d).

Dart shooting towards a target neural cell can generate transient pressure on the cell membrane upon impact, serving as an optomechanical nanotransducer for neural stimulation and modulation. To show the transient pressure effect of the dart with high precision, we performed additional experiments to shoot the dart toward different targets. As shown in Fig. 2i-I, the dart can be precisely shot on a polystyrene (PS) particle (900 nm in diameter). After hit by the dart, the particle was pushed forward by the dart with a distance of 15.5 µm within 0.1 s (Supplementary Video S3). While for a red blood cell, as shown in Fig. 2i-II, the cell membrane concaved after being hit by the dart, indicating the application of transient pressure on the cell membrane. To further show this transient pressure on cell membrane, numerical simulation was conducted. As shown in Fig. 2j, increasing pressure was exerted on the cell membrane as the velocity of the dart hitting it increased. The pressure linearly increases from 0 to a maximum of 516 pN μm^−2^ at 148 μm s^−1^. These results show that the pressure exerted on cell membrane can be controlled by adjusting the velocity of the dart hitting it. Previous study shows that a mechanical pressure of 228 pN μm^−2^ is sufficient for the opening of mechanosensitive ion channels (Piezo1 channels)^41^. Therefore, for stable control, in our experiments for neural stimulation and modulation, we controlled the dart with a shooting velocity of 120 μm s^−1^ hitting the cell membrane. In this case, the mechanical pressure exerted on the cell membrane was about 318 pN μm^−2^. Further experimental results show that as the velocity of the dart shooting toward the target cell was further increased to about 240 μm s^−1^, the dart can pierce into the cell membrane. At this state, the cell viability was not affected by the dart (Supplementary Fig. S9a). However, as the velocity was increased to about 280 μm s^-1^, the viability of the cell was affected (Supplementary Fig. S9b).

To directly verify that the bio-dart can generate mechanical interaction with the cell membrane of neural cells, we captured different views of a neural cell after hit by a dart using a confocal laser scanning microscope (details see in Materials and methods section in the Supporting Information). As shown in Fig. 2k, the dart (red fluorescence) was embedded in the cell membrane (green fluorescence). As indicated by fluorescence colocalization in Supplementary Fig. S10, a 3.6% overlap between the cell membrane and dart in space was observed, indicating contact between the dart tip and the cell membrane. This dart embedding in the cell membrane can also be observed from the SEM image (Supplementary Fig. S11). By flexibly manipulating the SMOFP, the dart can be flexibly shot to target regions of subcellular structures. As an example, Fig. 2l shows the targeted localization of dart on different subcellular parts (dendrites, soma, and axon) of the same cell (detailed targeting process see in Supplementary Fig. S12). This high-precision localization of bio-dart is very important for neural stimulation and modulation with sub-cellular precision^42^.

### Precision neural stimulation

**Fig. 3:**
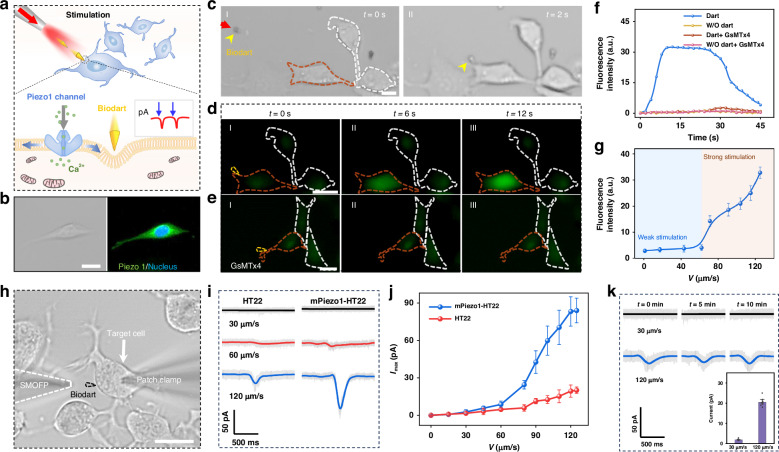
**Precision neural stimulation**. **a** Schematic illustration of precision neural stimulation by optomechanical bio-dart. The mechanical stimulation on cell membrane results in the activation of Piezo1 channel, with increasing in Ca^2+^ influx and inward current. **b** The bright field (left) and fluorescent (right) images of neural cell with Piezo1 channels identified in fluorescence green, and the nuclei in blue. **c** Microscopic images showing shooting of dart toward a single neuronal cell. Yellow arrow indicates the dart. **d**, **e** Fluorescent images showing intracellular Ca^2+^ response of a target single HT22 cell after stimulation. Cells in (**e**) were modified with GsMTx4. **f** Normalized fluorescence intensity of intracellular Ca^2+^ for neuronal cells with different stimulation as a function of time. **g** Fluorescence intensity change of stimulated cell as a function of dart velocity. **h** Microscopic image showing single-cell electrophysiological experiment for recording inward current of target stimulated neuronal cell using patch clamp. **i** Representative recorded inward current of stimulated HT22 cell and overexpressing Piezo1 HT22 cell with different stimulation velocity. The shading curves are the raw measured data, while the black, red, and blue curves are the fitting data. **j** Maximal inward current (*I*_max_) as a function of different stimulation velocity. **k** Inward current recording for repeated stimulation of HT22 cell, inset shows the quantitative analyses of the recorded maximal inward current of two different stimulation. Red curves show the boundary of target cell with stimulation, while white curve shows the neighboring cell without stimulation. The red and yellow arrows indicate 808 nm laser and dart, respectively. Scale bars: 20 µm

The precision neural stimulation is realized by the transient mechanical pressure exerted on the cell membrane by the optomechanical bio-dart, which subsequently activates the Piezo1 channels on the cell membrane, leading to an increase in cellular electrical signals (Fig. 3a). The activation of Piezo1 channel results in the influx of calcium ions and activation of the molecular pathway, subsequently leading to the increase of ATP generation in mitochondria. The Piezo1 channel is a mechanosensitive cation channel, which, upon mechanical stimulation, opens the channel proteins on the cell membrane, allowing extracellular cations such as K^+^, Na^+^, and Ca^2+^ to enter the cell. Amplified by gated channels, this process can generate enhanced electrical signal fluctuations^43^. To examine the expression of Piezo1 channels, we validated the presence of Piezo1 channels in HT22 cells, a mouse hippocampal neuron cell line selected as a neural cell model, through immunostaining. As shown in Fig. 3b, both bright field and fluorescence images of HT22 cells were captured. The fluorescence image reveals evident green fluorescence upon staining with Piezo1 antibody, indicating the expression of the Piezo1 channel in HT22 cells.

To show the precision stimulation of neural cells, as an example, Fig. 3c shows the targeted shooting and hitting of an optomechanical dart toward the subcellular position of a HT22 cell among a cell cluster. Supplementary Video S4 also shows another example of dart shooting toward a single neuronal cell. After shooting the dart to the cell, we monitored the transient calcium ions response upon mechanical stimulation within the cells using fluorescent Fluo-4 AM as an intracellular Ca^2+^ indicator. When neurons are stimulated and become active, intracellular Ca^2+^ levels can be 10–100 times higher than that at rest^44^. As shown in Fig. 3d, a significant increase of fluorescence signals was captured for the target cell after hit by dart, indicating the increase of Ca^2+^ influx, while no fluorescence changes were observed for the neighboring cells without dart action. These results indicate that the dart exhibits the capability for neural stimulation with single-cell precision. To further confirm that the increase of Ca^2+^ influx is indeed from the activation of the mechanosensitive channel Piezo1, we tested the Ca^2+^ influx using a typical mechanical channel inhibitor peptide GsMTx4. GsMTx4 can inhibit the activation of mechanosensitive channels by inserting into the compressed cell membrane and distorting the tension near the mechanosensitive channel proteins^45,46^. For HT22 neuronal cells pre-treated with GsMTx4, no enhancement of green fluorescence was observed under the stimulation of optomechanical dart (Fig. 3e). The real-time fluorescence intensity changes also verify this conclusion (Fig. 3f). These results indicate that the increase in Ca^2+^ influx after dart stimulation is indeed from the activation of Piezo1 channels. To quantify the mechanical stimulation effect of the dart, we stimulated cells with different hitting velocities. As shown in Fig. 3g, no significant change in Ca^2+^ fluorescence intensity was observed for mechanical stimulation by darts with hitting velocity below 65 μm s^-1^. We refer to this stimulation region as weak stimulation. For a velocity larger than 65 μm s^-1^ (strong stimulation), the fluorescence intensity of Ca^2+^ can be observed (Supplementary Fig. S13). Therefore, the transient mechanical pressure resulted from this hitting velocity serves as the activation threshold to open the Piezo1 channel, allowing Ca^2+^ to flow inward and causing an enhancement in Ca^2+^ fluorescence.

To further investigate the effects of optomechanical dart stimulation and activation on neurons at different hitting velocities, inward currents of HT22 cells were recorded using cell patch clamp to identify mechanosensitive currents induced by optomechanical dart stimulation (Fig. 3h). Inward current signal was captured when the dart hitting velocity was larger than 60 μm s^−1^ (±5 μm s^−1^) (Fig. 3i), indicating cell excitation. This threshold velocity was a little smaller than that in the recording of the fluorescence Ca^2+^ signal as shown in Fig. 3f. This is because the inward current recording is more sensitive than the fluorescence signal. As the hitting velocity was increased to 120 μm s^−1^, the peak current signal significantly was increased to 20 pA. The mechanosensitive inward current exhibits a duration of approximately 200 ms, and then returns to the resting current level. The native expression of Piezo1 channels in HT22 cells is typically very limited, which can impede the investigation of signal transduction. To further show the effect of Piezo1 channel on neuronal stimulation and activation by the optomechanical dart, as a comparison, we performed additional experiments using Piezo1 overexpressing HT22 cells via mPiezo1 plasmids transfection (mPiezo1-HT22 cells). As shown in Fig. 3i and Fig. 3j, the recorded inward current was significantly increased for mPiezo1-HT22 cells when stimulated with the same hitting velocity of dart. For example, an inward current of about 84 pA was recorded for the mPiezo1-HT22 cell with dart velocity of 120 μm s^−1^, much larger than that of a normal HT22 cell under the same stimulation (20 pA). Figure 3j also shows that the inward current of neuronal cells generated after stimulation by dart was increased with the increase in dart velocity, showing a clear mechanical stimulation dependence. We next investigated whether the dart can trigger neuronal activation repeatedly. Figure 3k shows the inward current of the same neuron cell upon repeated dart stimulation for three times with an interval of 5 min between each stimulation (images showing this repeated stimulation can be seen in Supplementary Fig. S14). Successful activation was recorded for each stimulation on the same neuron with the dart velocity of about 120 μm s^−1^, which confirmed the repeatability of neuronal stimulation by the optomechanical dart.

It should be noted that in addition to exerting transient pressure on the cell membrane to activate the Piezo1 channel for neuronal stimulation and activation, our bio-darts can also serve as a drug loading tool for other neuronal-related applications. In this case, by delivery the loaded drug to the target cell via shooting the dart toward a target cell, multifunctional neuronal regulation can be realized. For example, the dart can also be used for neuronal silencing. In this case, HT22 cells were initially treated with Piezo1 channel agonist Yoda1 so that the cells were in an activation state. The darts were loaded with mechanical channel inhibitor GsMTx4. After precisely shot to the target cell (cell A in Supplementary Fig. S15a), GsMTx4 was released from the dart to the cell, and obvious Ca^2+^ fluorescence intensity decrease was observed compared with the neighboring cell without dart treatment (Supplementary Fig. S15b–d).

### Neuronal modulation with sub-cellular precision

**Fig. 4:**
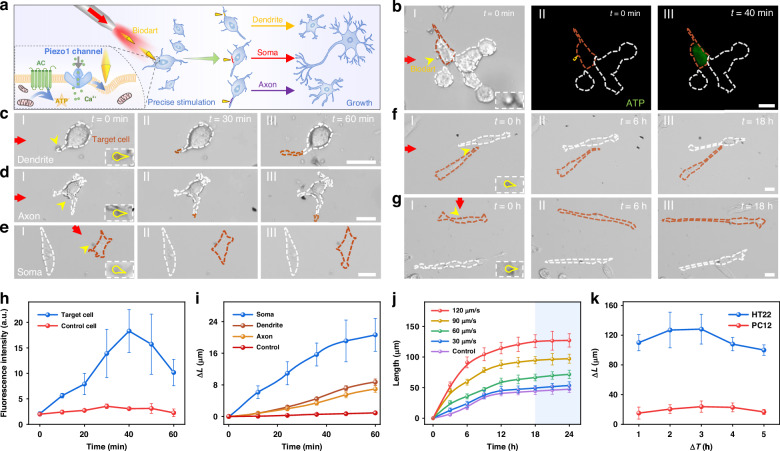
**Precision neuronal modulation and controlled neuronal growth**. **a** Schematic illustration of neuronal modulation and growth via optomechanical bio-dart with precision down to subcellular neuronal structures. Inset shows the activation of intracellular molecular pathway for increased ATP generation after dart stimulation. **b** Intracellular ATP response of a target HT22 cell after stimulation with dart. Panel I: bright field image showing dart stimulation of a target cell. Panels II, III: fluorescent images showing ATP response. Microscopic images showing precision modulation and short-term growth of subcellular neuronal structures (**c**) dendrite, (**d**) axon, and (**e**) soma. Red arrow indicates the direction of dart shooting, yellow arrow indicates the position of dart on target position of a neuronal cell, red curves on cell boundary shows the outgrowth of target cell. Microscopic images show long-term growth of target single neuronal cell after stimulation by dart with different velocities of (**f**) 30 µm s^−1^ and (**g**) 120 µm s^−1^. Red curves show the boundary of target cell with stimulation, while white curve shows the neighboring cell without stimulation. **h** Fluorescence intensity of ATP signal as a function of time after stimulation of soma. **i** Growth length of intracellular neuronal structures of HT22 cell as a function of time after stimulation. **j** Long-term growth length of HT22 cell as a function of time for dart stimulation with different velocities. Blue region indicates stationary phase of neuronal cell growth. **k** Average growth length as a function of stimulation interval for two different neuronal cell types. Scale bars: 20 µm. The statistical data in **h**–**k** are concluded based on 20 cell repeats, the error bars are standard error of the mean

In addition to the precision neural stimulation using our optomechanical bio-darts, the mechanical stimulation of the dart can further result in neuronal modulation and subsequent growth with sub-cellular precision. As shown in Fig. 4a, precise stimulation can be applied to subcellular compartments of neuronal cells, such as soma, axons, and dendrites, through controlled shooting of darts. This precision stimulation at different sub-cellular structures of neuronal cells can promote the modulation of neuronal growth. The activation of Piezo1 channel results in the increase of Ca^2+^ influx after neuronal stimulation, and intracellular molecular signaling pathways can further be activated. For example, cyclic adenosine monophosphate (cAMP)-dependent pathway, also known as adenylate cyclase (AC) pathway, is one of the most important molecular signaling pathways in neuronal cell growth and can be activated by various extracellular stimuli^47,48^. The increase in intracellular Ca^2+^ concentration can form a complex with calmodulin, leading to the activation of AC pathway. AC is a key regulatory enzyme that catalyzes the conversion of Adenosine Triphosphate (ATP) into cAMP. cAMP molecule is a secondary messenger molecule that can activate protein kinase A (PKA)-dependent neuron differentiation pathway. The stimulation of neuronal cell by our optomechanical dart can further induce the modulation of neuronal cell differentiation and growth through the cAMP-dependent pathway (Figs. 1b and 4a). ATP generation is a downstream effector of Piezo1 channel activation. To show the ATP change after neuronal stimulation by dart, pCMV-AT1.03, an ATP fluorescent (green) probe, was introduced into HT22 cells. Figure 4b shows the fluorescence images of ATP generation of HT22 cells after stimulation by dart (120 μm s^-1^). Obvious green fluorescence for ATP was observed 40 min after stimulation for the target cell with dart stimulation (Fig. 4b-III, h), while neighboring cells without stimulation showed no fluorescence change. These results indicate that mechanical stimulation increases ATP generation in neuronal cells. ATP can provide energy to activate the cAMP pathway, which can further facilitate neuronal modulation and neuronal growth.

To show the precision neuronal modulation capability of the dart, single dart was precisely shot toward different subcellular structures of HT22 cells. We find that after further culturing of the stimulated cells, a remarkable outgrowth of the stimulated sub-cellular parts was observed while neighboring non-stimulated cells show no observable changes in this observation period. Figure 4c–e and Supplementary Fig. S16, as some examples, show the growth of stimulated sub-cellular parts of HT22 cells after optomechanical stimulation by dart (120 µm s^−1^). Stimulation with darts on soma, axons, and dendrites shows average elongations of neurites at 20.6 ± 4 μm, 8.7 ± 0.7 μm and 7 ± 0.8 μm (20 cells repeats), respectively, within one hour (Fig. 4i). In contrast, cells without mechanical stimulation show no significant neurite growth within one hour. The results indicate that optomechanical stimulation of darts can effectively induce in-situ growth of neuronal cells with sub-cellular precision. Meanwhile, among the neuronal sub-cellular structures, soma is more easily to be affected by mechanical stimulation and shows more significant growth responses, whereas the less susceptible axons and dendrites respond transiently. This is because the distribution of Piezo1 channel is highly related to the membrane curvature^49^. The concentration of Piezo1 channels on highly curved membrane structures, such as the membrane filopodia, dendrite, and axon, is less than less curved membrane structures, such as the soma. This can also be observed from the fluorescent image showing the distribution of Piezo1 in Fig. 3b. These results show that targeting of the soma by our optomechanical dart can result in a much more effective opening of the Piezo1 channels for neuronal stimulation and modulation.

After verifying the ability of darts to promote the growth of neuronal cells with sub-cellular precision in the short term, we further investigate the long-term modulation capability of neuronal cells within 24-hour culturing. Previous results show that our darts can repeatedly trigger neural activation. To get the long-term modulation of neuronal cells, repeat stimulation with different time intervals was performed on two standard neuronal cells (HT22, PC12). It was found that repeat stimulation at an interval of 3 hours can achieve optimal growth effect (Fig. 4k, dart velocity: 120 μm s^−1^). Therefore, subsequent experiments for long-term neuronal modulation were conducted with a 3-hour interval of repeat stimulation. With dart stimulation at a low velocity, for example, 30 μm s^−1^, applied on HT22 cells, only natural growth was observed on the cell (Fig. 4f and Supplementary Fig. S16). No significant increased growth was observed when compared to the neighboring cell without dart stimulation. In contrast, with a high-velocity stimulation on the neuronal cell (120 μm s^−1^), a significant increase in the growth rate of the stimulated cell was observed when compared with the neighboring cell without stimulation (Fig. 4g and Supplementary Fig. S16). As shown in Fig. 4j, statistical analysis on the length of neuronal cells after stimulated with dart at different velocities reveals that neural cells exhibit rapid growth within the first 12 h after stimulation, followed by a gradual deceleration until reaching a stable phase at around 18 h. This growth rate was increased with the increase in stimulation velocity. The results also indicate that the stimulation velocity threshold of the dart on HT22 cells is 30 μm s^−1^, and successful promotion of neuronal cell growth can be observed with the dart velocity larger than this threshold value. To show the applicability of the optomechanical darts for neuronal modulation, the modulation of another neuronal cell, PC12 was also performed. As shown in Supplementary Fig. S17, increased growth of stimulated PC12 cells was also observed. However, the average increased growth rate of PC12 cells is smaller than that of HT22 cells. This difference may be attributed to the inherently slower proliferation rate of PC12 cells. All the results show that our optomechanical bio-darts are capable of neuronal modulation with high precision.

In addition to the stimulation and modulation of neurons with Piezo1 channels, the drug loading and delivery capability of our dart can also be used for the stimulation and modulation of neurons without Piezo1 channel. As an example, Supplementary Fig. S18 shows the stimulation of neurons without Piezo1 channel (F11 dorsal root ganglion cells and SH-Sy5y neuroblastoma cells) by loading the dart with β-nerve growth factor (β-NGF). After precision shooting of the dart to the target cell, the loaded β-NGF was released to the target cell, and the treated cell shows a higher growth rate than the neighboring cells without dart treatment.

### High-throughput and in vivo neural stimulation

For a real in vivo environment, the density of neurons is generally very high. To realize the high-throughput of neural stimulation at this high-density environment, the number of darts that can be shot out toward multiple cells should be increased. In fact, our method can also be used for high-throughput dart shooting and neural stimulation. To demonstrate this capability, we used a flat multi-mode optical fiber probe (MMOFP) without tapering to replace with the flat-end tapered SMOFP. The diameter of the MMOFP core is 62.5 μm (Supplementary Fig. S19b), which is much larger than the end of the flat-end tapered SMOFP we used for single neuronal stimulation. MMOFP with this increased size can be used for light delivery with an increased area. This larger area for light delivery can result in an increased throughput for dart shooting by optical force at one time. The calculated optical force exerted on bio-darts using a MMOFP is shown in Supplementary Fig. S20. In addition, by moving/scanning the slantly positioned fiber (schematically shown in Supplementary Fig. S19a), hundreds of darts can then be shot out at different locations (Supplementary Fig. S19c), and the throughput of neuronal stimulation can thus be increased (Supplementary Fig. S19d). As shown in Supplementary Fig. S19e, using the flat end MMOFP with an optical power of 200 mW, more than 20 darts can be shot toward HT22 neuronal cells at *t* = 20 s. The velocity of darts at different positions of the light irradiation from the MMOFP is shown in Supplementary Fig. S20d. By scanning the MMOFP, darts at different positions can be shot toward more cells. At *t* = 60 s, about 25 cells were shot by darts. After shot with darts, high-throughput neuronal stimulation can be realized. As shown in Supplementary Figs. S21 and S22, increased Ca^2+^ signals were observed for more than 20 neuronal cells within a minute, indicating the large-scale neuronal stimulation. These results show that our method can also be used for high-throughput neuronal stimulation, which is demonstrated using other light-based technologies such as holographic-based two-photon optogenetics^50^. It should be noted that, for a dense cell environment, if multiple darts are irradiated by the MMOFP, then multiple cells can be simultaneously shot by the darts. However, by decreasing the dart concentration with only a single dart in the light irradiation region of the MMOFP, this MMOFP can also be manipulated to deliver the dart toward a target cell for single specific cell stimulation.

**Fig. 5:**
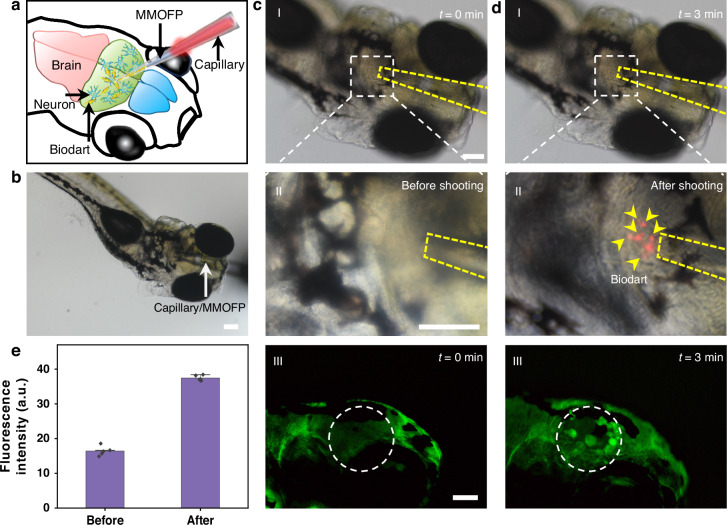
**In vivo neural stimulation with optomechanical bio-darts**. **a** Schematic illustration of neuronal stimulation by dart in larval zebrafish. **b** Microscopic image showing capillary/MMOFP inserting into the head of larval zebrafish. Neural stimulation (**c**) before and (**d**) after dart shooting toward brain tectum neurons. Panel I: bright field microscopic image showing capillary/MMOFP inserting; panel II: enlarged view of the target brain tecum, yellow curve indicates the capillary/MMOFP, yellow arrows indicate the Cy5-labeled darts; panel III: Intracellular Ca^2+^ response (green) of the neurons at the target region (white circle indicated) of the brain tectum before and after stimulation with darts. **e** Normalized fluorescence intensity of intracellular Ca^2+^ signal for neurons at the target region of brain tecum before and after stimulation. Scale bars: 100 µm

Our method can also be translated into the dense environment of the brain for in vivo neural stimulation. To realize this, we replaced the flat-end tapered SMOFP to a flat-end tapered MMOFP, with a diameter of the fiber end being 40 μm. In this case, the effective area of the optical scattering force is greatly increased, and multiple darts can then be shot out by the optical fiber toward a dense environment. We demonstrated the in vivo neural stimulation based on a GCaMP6s transgenic larval zebrafish (2 dpf), where the calcium activity in the neurons can be observed via green fluorescence. As schematically shown in Fig. 5a, dart solution (Cy5 labeled for visualization) was injected into a tapered borosilicate glass capillary (inner diameter at the end: 45 μm, outer diameter: 49 μm), and a MMOFP was then inserted into the capillary. For a better targeting and manipulation, the capillary was carefully positioned using a micromanipulator under a fluorescent microscope. The capillary was punctured into the skin of the head until superficially piercing the brain (Fig. 5b, c). This process took about 2 min. After capillary/fiber insertion, fluorescent images of the insertion region were taken to compare with the fluorescent signal of neural cells before insertion. No fluorescence changes were observed near the capillary insertion site. 1 min later, darts were then shot toward the neurons in brain tectum with an optical power of 200 mW launched into the MMOFP (Fig. 5d). This process took about 30 s. The neurons were stimulated due to the transient pressure on cell membrane by darts, and increase in the Ca^2+^ fluorescence intensity was observed (Fig. 5dIII). These results justify that the neural stimulation is from the dart and not from the capillary insertion. Compared with the cells without dart shooting, an average fluorescence intensity increases of about 128% was captured (Fig. 5e), indicating the neural stimulation and activation in vivo. It should be noted that the darts used for neural stimulation were not removed after the stimulation experiment. Due to the very small number of darts (about 20), the darts remain in the zebrafish brain would not affect the brain function (details see in Supplementary Fig. S23 and corresponding descriptions in the Supporting Information).

## Discussion

In summary, we demonstrated an optomechanical bio-dart designed for precise neural stimulation and modulation at the subcellular level. Such bio-dart can be shot out and directly navigated by optical scattering force for targeted stimulation of neuronal cell with transient membrane mechanical pressure. This precision stimulation activates the mechanosensitive Piezo1 ion channels on the neuronal cell membrane, subsequently resulting in the influx of Ca^2+^, activation of intracellular molecular pathways, and the increased generation of mitochondrial ATP. Precision neuronal modulation was further realized by this optomechanical dart, and controlled growth of single neuronal cells as well as subcellular neuronal components, such as dendrite, soma, and axon, was realized. This bio-dart can also serve as a drug delivery tool for multifunctional neural stimulation and modulation. Importantly, in vivo neural stimulation in larval zebrafish was also realized using our darts.

Although optogenetics is currently the most popular and important technique for neuronal modulation with precise spatiotemporal control, optogenetics relies on genetic modification of the cells for light-sensitive protein expression. This genetic modification also exhibits some other recognized drawbacks, such as complex genetic modifications, unstable gene expression of modified genes, and unintended off-target effects^25,51^. The complexity of genetic modification operations makes it complex and time-consuming, and the cost can be relatively expensive. Our work introduces a nongenetic optomechanical neural modulation approach, which preserves the benefits of optical modulation such as high spatiotemporal resolution, while circumventing the limitations associated with optogenetics. This strategy does not alter the neuronal cells, but instead uses the transient optomechanical stimuli from bio-darts that are shoot to the cell membrane. It is worth mention that the material used in this study is sunflower pollen, which is readily accessible and cost-effective. The preparation of bio-darts is quite convenient and low-cost as compared to genetic modification process in optogenetics, which makes our strategy more accessibility to researchers with limited resources. Although optical tweezers can be used for particle and cell manipulation/measurements with high spatial and temporal control^52^, direct stimulation and modulation of neurons is generally difficult. For neuronal stimulation and modulation using optical tweezers, a trapped microbead is often required to indirectly exert force onto the neuron^53^. However, the size of the manipulated particles is generally several micrometers, which limits the stimulation and modulation precision. While for our darts, the size of the dart tip is only 170 nm, which is much smaller than that of optical tweezers-manipulated microbead. This smaller size enables our dart for high precision neuronal stimulation and modulation with sub-cellular precision. which can easily target the subcellular region of a single neuronal cell, generating transient mechanical pressure for neuronal stimulation with sub-cellular precision. By utilizing this transient mechanical pressure, we demonstrate precise activation of single neuronal cells without affecting neighboring cells. Most importantly, the capability of modulation of a single neuronal cell, with even precision down to subcellular neuronal structures, such as neuronal dendrites, axons, and soma, holds great promises for a better understanding of the mechanism of neuronal growth, signaling, plasticity, and dysfunction. Although our optomechanical bio-darts can be used for the sub-cellular neural stimulation and modulation, the effective stimulation is relied on the transient pressure on the cell membrane generated from the tip of the bio-dart. However, currently we are not able to precisely control the orientation of the bio-dart (i.e., tip or base forward) shooting toward the target region of the cell, which limits the stimulation efficiency of the bio-darts. Fortunately, this limitation can be overcome via controlled orientation of the bio-darts by modification of the bio-darts with magnetic nanomaterials. In this case, the bio-darts can be controlled with the tip shooting toward the target cell membrane for high-efficiency neural stimulation with a magnetic field.

## Materials and methods

Details of the materials and methods can be seen in the Supporting Information.

## Supplementary information

Supporting Information

Orientation and acceleration of Bio-dart

Bio-dart shooting in different bio-microenvironments

Bio-dart shooting towards different targets

Bio-dart shooting toward a single neuronal cell
