# Supplementary material for: Neural stimulation and modulation with sub-cellular precision by optomechanical bio-dart
Source: Light Sci Appl. 2024 Sep 19;13:258. doi: 10.1038/s41377-024-01617-9 (PMC11413014; doi:10.1038/s41377-024-01617-9)
Supplement: Supplementary file 1 — Supporting Information [file 41377_2024_1617_MOESM1_ESM.docx]

**Supporting** **Information for**

**Neural stimulation and modulation with sub-cellular precision by optomechanical bio-****dart**

Guoshuai Zhu^1^, Jianyun Xiong^1^, Xing Li^1^, Ziyi He^1^, Shuhan Zhong^1^, Junlin Chen^2^, Yang Shi^1^, Ting Pan^1^, Li Zhang^2^, Baojun Li^1*^ and Hongbao Xin^1*^

^1^ Guangdong Provincial Key Laboratory of Nanophotonic Manipulation, Institute of Nanophotonics, College of Physics and Optoelectronic Engineering, Jinan University, Guangzhou 511443, China

^2^ Key Laboratory of CNS Regeneration (Ministry of Education), Guangdong-Hong Kong-Macau Institute of CNS Regeneration, Jinan University, Guangzhou 510632, China

^*^To whom correspondence may be addressed

**Email:** [baojunli@jnu.edu.cn](mailto:baojunli@jnu.edu.cn), [hongbaoxin@jnu.edu.cn](mailto:hongbaoxin@jnu.edu.cn)

**This Supporting Information file includes:**

**1. Materials and methods**

**2. Supporting Figures**

Fig. S1. to S23

**3. Description of Supporting videos**

Video S1.to S4

**Materials and methods**

**Bio-dart preparation:** Sunflower pollen grains (SPGs) were defatted to remove pollenkitt. Bee pollen granules were refluxed in acetone for 1 h under magnetic stirring (50 °C, 300 rpm). Next, the pollen sample was hydrated with deionized water (50 °C) with magnetic stirring for 30 min. Afterward, defatted SPGs were suspended in KOH (10% (w/v) to remove the internal core substances by heating it at 80 °C under 400 rpm. At this time, hollow SPGs was collected. Next fresh 10% (w/v) KOH was added to the pollen sample, followed by oscillating used the ultrasonic cleaner for 30 min. The resulting pollen dart was collected by the last step of centrifugation followed by the supernatant removal, then stored at 4 °C for further characterization. In the experiment, pollen darts were freeze-dried under -80 ℃ conditions to obtain dart freeze-dried powder. When used, it is dissolved in PBS and sterilized with ultraviolet radiation. For the demonstration of the drug-loading capability of the darts, the dart solution (in PBS) was mixed with either nerve growth factor (β-NGF) or a neural silencing drug (GsMTx4), and the mixture was slowly stirred for 8 hours at room temperature. The mixture was then centrifuged and collected, followed by three washes with PBS to remove any excess drugs.

**Methods for fabrication of SMOFP:** The optical fiber probe was fabricated from a commercial single-mode optical fiber. The buffer and polymer jacket of the fiber were stripped off with a fiber stripper to obtain a 2 cm length of bare fiber. Before being heated, the fiber was sheathed with a steel capillary (inner diameter = 0.9 mm, wall thickness = 0.12 mm, length = 300 mm) to prevent the fiber from bending and breaking. The bare fiber outside the capillary was heated for about 100 s until it reached its melting point. Then, the fiber was drawn through a heating zone of approximately 5 mm at a speed of about 3 mm s^-1^, which then gradually tapered off, causing its diameter to decrease from 125 to 15 μm over a length of approximately 2 mm. The optical fiber was then removed from the flame and cooled to room temperature for 2 min. Finally, the tapered region of the optical fiber was cut by a fiber cleaver to obtain a smooth end surface. The final diameter of the fiber probe can be modified by controlling the heating time and drawing speed.

**Experimental setup**: To achieve neuronal stimulation and modulation with sub-cellular precision by dart, a laser beam of 808-nm wavelength from the laser source (VLSS-808-B, Shanghai Conet Laser Technology Co., LTD, China) was launched into the SMOFP. The SMOFP sheathed by a glass capillary can be flexibly manipulated by the six-axis manipulator in 3D, thus the SMOFP can be manipulated in 3D. To get a better manipulation result, the dart suspension above a glass slide can be further positioned by an *x-y* manual translation stage (resolution: 100 nm). Combining the manipulation of the six-axis manipulator and translation stage, the driven dart can be flexible and precisely manipulated in 3D and thus the dart can be precisely controlled to shoot toward the target region of a neuronal cell by optical scattering force along the optical axis of the fiber probe.

**Cell culturing:** Hippocampal neuronal cell lines (HT22) was cultured in Dulbecco’s Modified Eagle Medium (DMEM) supplemented with 4500 mg L^-1^, 10% FBS and 1% Penicillin/streptomycin (Pen/strep) in a 5% CO_2_ incubator at 37 °C. Cells were seeded 24 h into a Petri dish with a slide to reach 80-90% fusion degree of the cells on the slide before the experiment.

**Cell viability test:** Hippocampal neuronal cell viability was tested using dual-fluorescent calcein-AM/PI (purchased from Jiangsu KeyGEN BioTECH Corp., LTD, China). In the experiments, 2 µL AM and PI dyes were added to co-cultured cells for 25 min. Subsequently, the sample was washed twice with PBS buffer solution. For living cells, Calcein-AM can react with esterases, and then strong green fluorescence was emitted for live cells. PI cannot pass the living cell membrane but can reach the nucleus through the disordered region of the dead cell membrane. Therefore, the DNA double helix structure in the cell was bound with PI, so red fluorescence was emitted for dead cells.

**Cell proliferation assay:** Dispense 100 μL of cell suspension (1500 cells/well) in a 96-well plate. Pre-incubate the plate for 24 hours in a humidified incubator (e.g., at 37 °C, 5% CO_2_). Then, add 10 µL of various concentrations of dart to be tested to the plate. Incubate the plate for an appropriate length of time (e.g., 1, 3, 5 days) in the incubator. Add 10 µL of CCK-8 solution to each well of the plate. Be careful not to introduce bubbles to the wells, since they interfere with the O.D. reading. Incubate the plate for 2.5 hours. Measure the absorbance at 450 nm using a microplate reader.

**Plasmid transfection:** The plasmid pcDNA3.1-mPiezo1-IRES-eGFP was purchased from Ke Lei Biotech Co., Ltd. (Shanghai, China). HT22 cells were seeded into 35 mm culture glass-bottomed confocal dishes at 5 x 10^5^ cells per dish. The next day, cells were transfected using the Lipofectamine 2000 kit (Invitrogen). 3 µg plasmid and 10 µL of Lipofectamine 2000 were complexated in Opti-MEM medium (Gibco) according to the manufacturer’s instructions and added to the cells. The cells were used for further experiments 24 hours later.

**Piezo1 labeling:** Cells were fixed using 4% paraformaldehyde and permeabilized using 0.5% Triton X-100, and all washes were done with 1X PBS. Cells were blocked using 5% BSA and incubated overnight in rabbit anti-piezo1 (Proteintech) antibodies diluted in PBS. Secondary antibody (Alexa Fluor 488) incubation was performed the next day, diluted in 2% BSA in PBS for one hour at room temperature. Cells were washed, coverslips dried, and mounted on glass slides using small drops of Prolong Glass Antifade Mountant with NucBlue (Life Technologies) and allowed to cure overnight at room temperature. All steps from the secondary antibody incubation onwards were performed in the dark. Coverslip edges were then sealed using transparent nail enamel and imaged using a confocal laser scanning microscope (TCS SP8, Leica).

**Calcium imaging and analysis:** HT22 are washed with serum-free DMEM/F12 before the addition of calcium indicator. Fluo-4 AM (4 μL, 2 µM) was incubated at 37 °C for 30 min. After discarding the supernatants, DEP medium (0.3 Osm sucrose solution with 1% FBS and 20 mM HEPES, pH 7.4) was added for 30 min at 37 °C. After the dart were introduced, the dart was guided to a predetermined target cell under 808 nm light. After stimulating the target cell, the calcium ion fluorescence signal was captured by a Nikon Ti2-A inverted fluorescence microscope. ImageJ was used to analyze the intracellular Ca^2+^ level.

**Electrophysiology of neuronal signal:** Whole-cell currents were recorded using borosilicate glass pipette with a resistance of 2-5 MΩ filled with intracellular solution containing (in mM): 10 NaCl, 140 KCl, 1 MgCl_2_, 5 EGTA and 10 HEPES. Briefly, we used a manual micromanipulator to move vertically the patch clamp pipette in contact with the cell membrane about 5 μm from the mechanical stimulation site. After reaching a seal between the membrane and the electrode of more than 1 GΩ resistance, we applied a gentle suction to break the membrane patch in order to enter in whole-cell configuration. Then, we proceeded with the simultaneous cell mechanical stimulation with velocity in the range of 0-120 μm s^-1^ and the characterization of the responses in the whole-cell configuration. For all experiments, the cells were bathed in an extracellular solution containing (in Mm) :140 NaCl, 2.8 KCl, 1 MgCl_2_, 2 CaCl_2_ and 10 HEPES and maintained at a holding potential of -70 mV.

**Confocal microscopic imaging:** The nucleus and cytoskeleton of the HT22 cells and the dart were stained in experiments to characterize the position relationship between HT22 cells and darts. The cells were washed three times with PBS to remove unadhered cells, and serum-free DMEM/F12 medium and phalloidin-TRITC (0.5 μg mL^-1^) solution was added to the culture dish for 30 min to stain the cytoskeleton. Finally, the nuclei were stained by adding 5 μg mL^-1^ Hoechst to the dishes and incubating for 5 min. After the dart were introduced, the dart was guided to a predetermined target cell under 808 nm light. After stimulating the target cell, the fluorescence signal was captured by a confocal laser scanning microscope (TCS SP8, Leica). The image was obtained via z-stack frame rate is 28 fps, step size is 300 nm, and imaging resolution is 180 nm. The overlap was concluded from the colocalization of the red fluorescence of dart and the green fluorescence of neuron cell, which was obtained using the plug-in (Coloc 2) in the software ImageJ.

**Characterization:** Scanning electron microscopy (SEM) of the dart were acquired by a Hitachi (SU1000) at an operating voltage of 15 kV. For the characterization of the dart, a drop of the solution was placed on a silicon wafer. For the characterization of the HT22 cells after mechanical stimulation, the coverslips with HT22 cells and dart were washed three times with PBS, and then 2 mL of 2.5 vol % glutaraldehyde PBS solution (pH 7.2) was added and kept at 4 °C overnight. After that, the coverslips were washed twice to remove glutaraldehyde and underwent the dehydration process by the immersion into ethanol solution of different concentrations of 30, 50, 75, 90, and 100% for 15 min stepwise. The cell sample then was dried in a vacuum oven overnight. Then the coverslips with HT22 cells and dart can be detected by SEM after the vacuum gold coating.

**In vivo neuronal stimulation:** All in vivo experiments were performed in compliance with the Laboratory Animal Ethics Committee of Jinan University. The used transgenic line of larval zebrafish (2 dpf) was obtained from the Nanjing Eze-Rinka Biotechnology Co., Ltd. (Nanjing, China). The larval zebrafish was Tg (actb2: GCaMP6) for visualization of the Ca^2+^ through fluorescence labeling in vivo. According to standard procedures, the zebrafish were maintained in a clean tank and cultured with a 14 h light/10 h dark cycle at 28.5 °C according to standard procedures. The larval zebrafish were anaesthetized by the incubation with MS-222 (0.08 mg mL^-1^, 5 min) and fixed on agarose slices. Then the dart (in PBS, 1 mg mL^-1^, Cy5 labelled for visualization) was loaded into a tapered borosilicate glass capillary, and the flat-end tapered multi-mode optical fiber was then inserted into the capillary. the capillary was carefully positioned using a micromanipulator under a fluorescent microscope. The capillary was punctured into the skin of the head until superficially piercing the brain. Darts were then shoot toward the neurons in brain tectum with an optical power of 200 mW launched into the fiber.

**Quantification and statistical analysis:** Origin software was used for all statistical analysis. All central tendency values are mean and error bars shown are standard deviation of the mean. In all case, at least three independent experimental repeats were carried out for each condition. Electrophysiological data were processed, analyzed, and baseline corrected with Clampfit 10.3 (Molecular Devices).

**2. Supporting Figures**


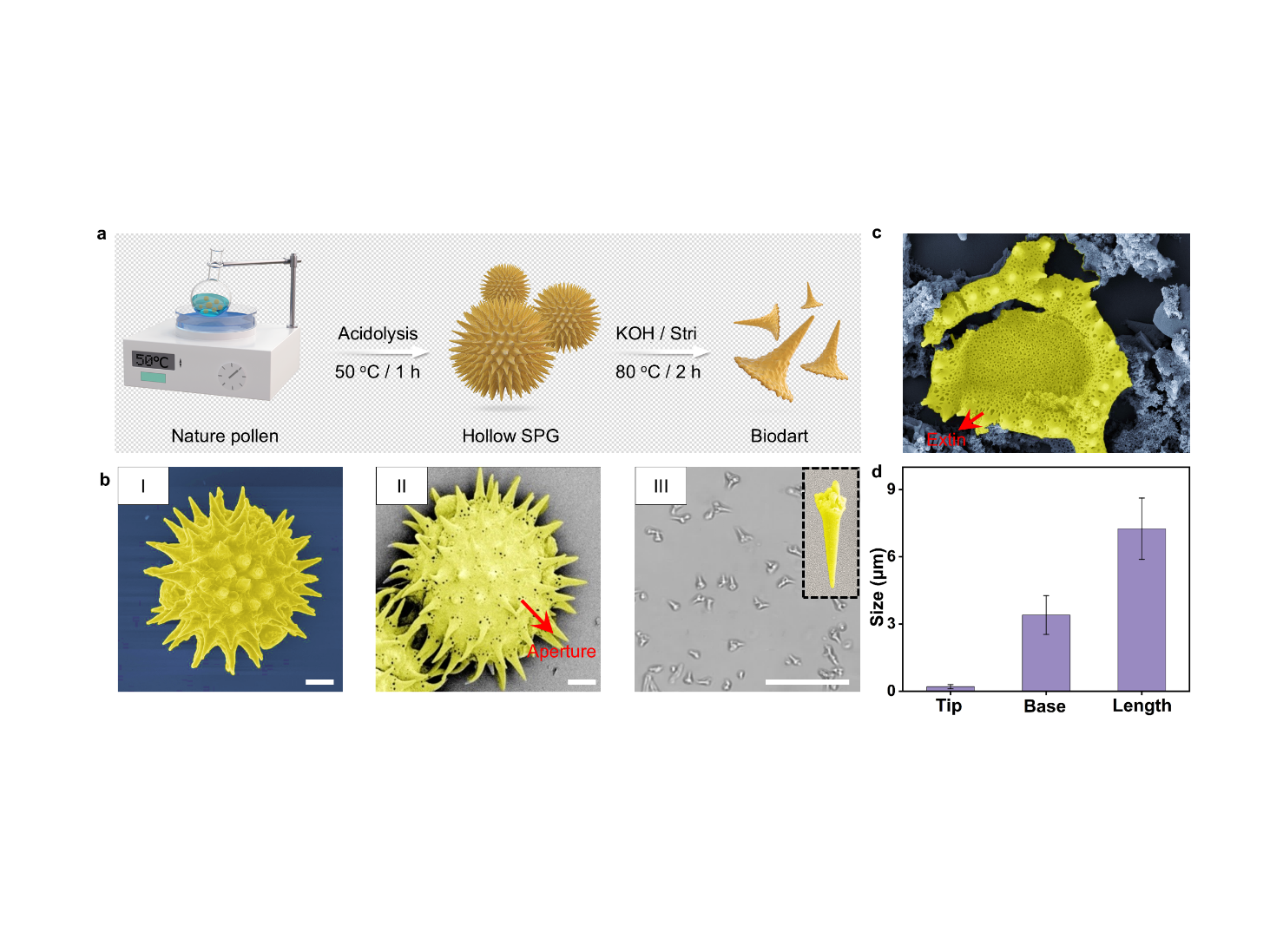


**Fig. S1.** **Preparation and characterization of bio-dart.** (**a**) Schematic illustration of demonstrating the preparation of dart. (**b**) SEM images of (I) natural SPGs, (II) Hollow SPGs after acidolysis and (III) Darts. (**c**) SEM images of the pollen exine. (**d**) Size of darts based on over 100 repeats. The value of the diameters is 0.17 μm, 3.1 μm and 7.5 μm, respectively. The pollen structures are pseudo-colored. Scale bars:20 μm.

As shown in the schematic diagram (Fig. S1a), the preparation sequence of darts involves three main steps: removing the internal cytoplasm by acid hydrolysis to obtain hollow pollen grain shells, softening the pollen shell under alkaline conditions and finally obtaining darts through ultrasonic vibration. The internal and surface of natural SPG are filled with cytoplasm and lipid compounds, respectively (Fig. S1b). Therefore, the tip of the natural SPG surface is not clearly visible. After acid hydrolysis treatment with phosphoric acid, substances inside the cytoplasm and lipid compounds attached to the surface were successfully removed. The SEM images clearly show that the treated pollen grains have a sea urchin-like shape (Fig. S1bll, average size: 35 ± 4.2 μm) with distinct nano peaks and hollow cavities inside (Fig. S1c). Scanning electron microscopy (SEM) analysis revealed that they possess an average size of 7.5 μm in length, 3.1 μm in width, and a tip diameter of 170 nm (Fig. S1d). Finally, we successfully prepared a large number of monodisperse darts.


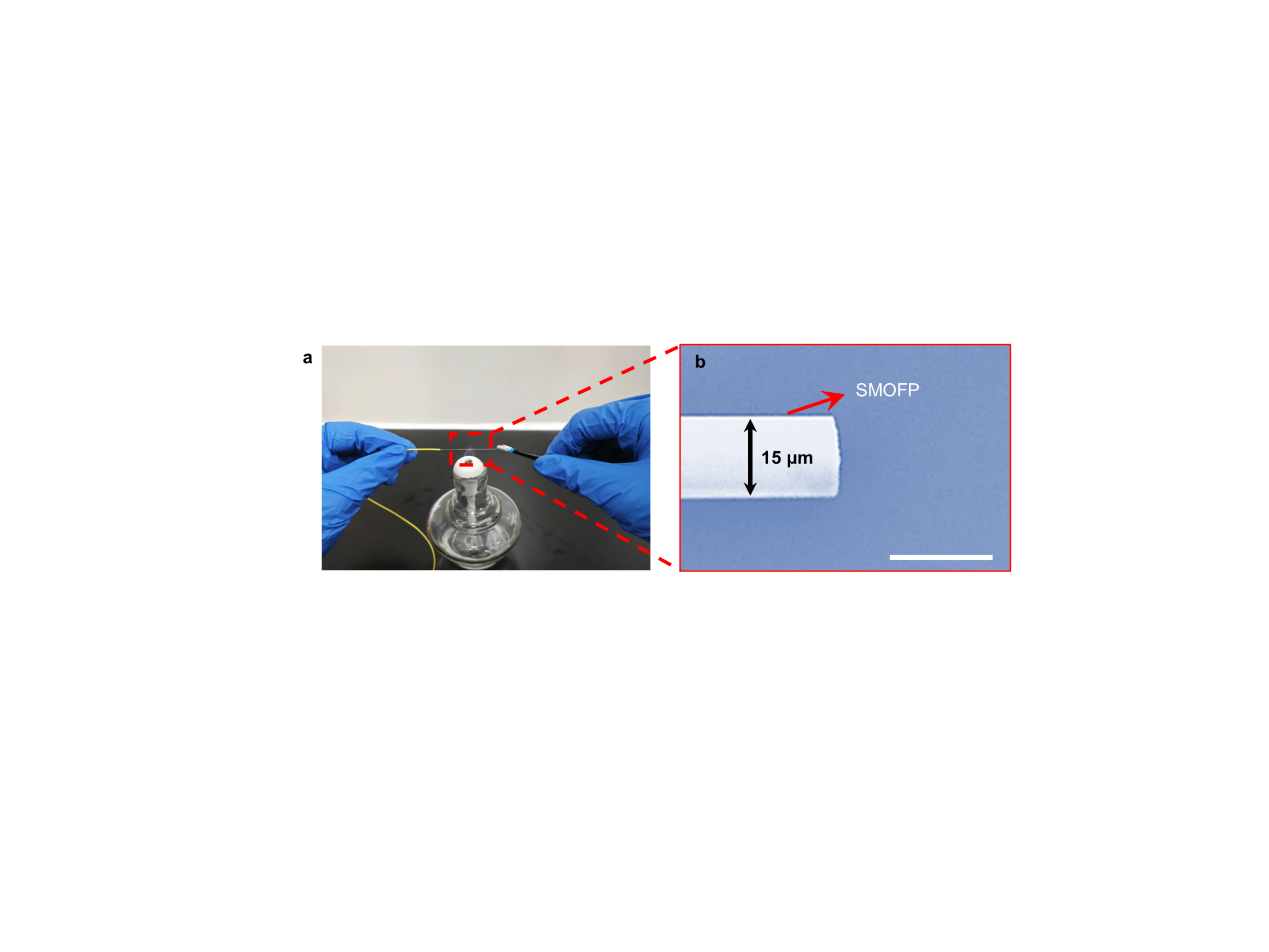


**Fig. S2. Preparation of the SMOFP.** (**a**) Preparing optical fibers using the method of fusion tapering. (**b**) Scanning electron microscope image of SMOFP. Scale bar: 20 μm.


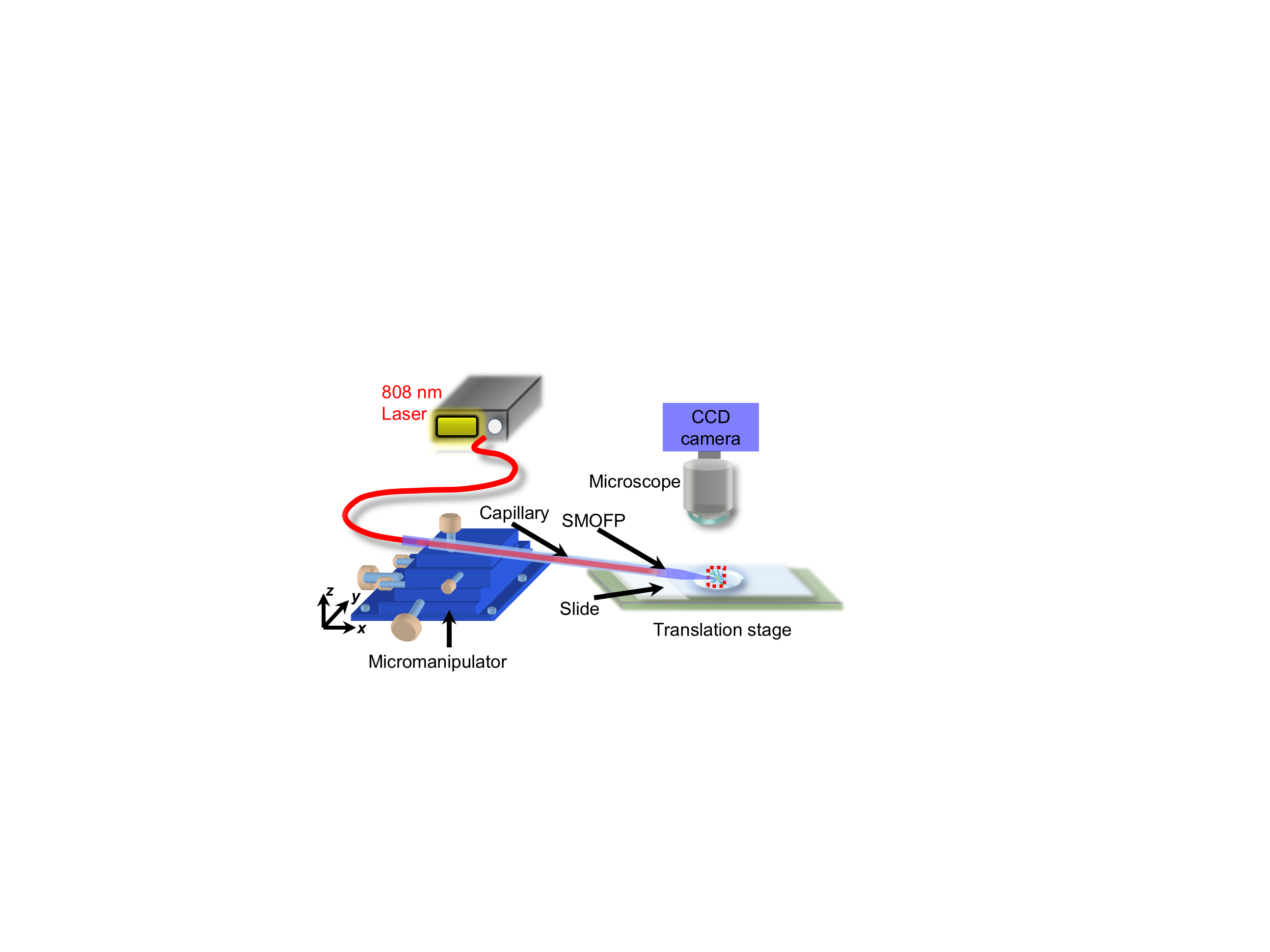


**Fig. S3. Schematic illustration of experimental setup.** The SMOFP is sheathed by a glass capillary and manipulated by a six-axis micromanipulator.


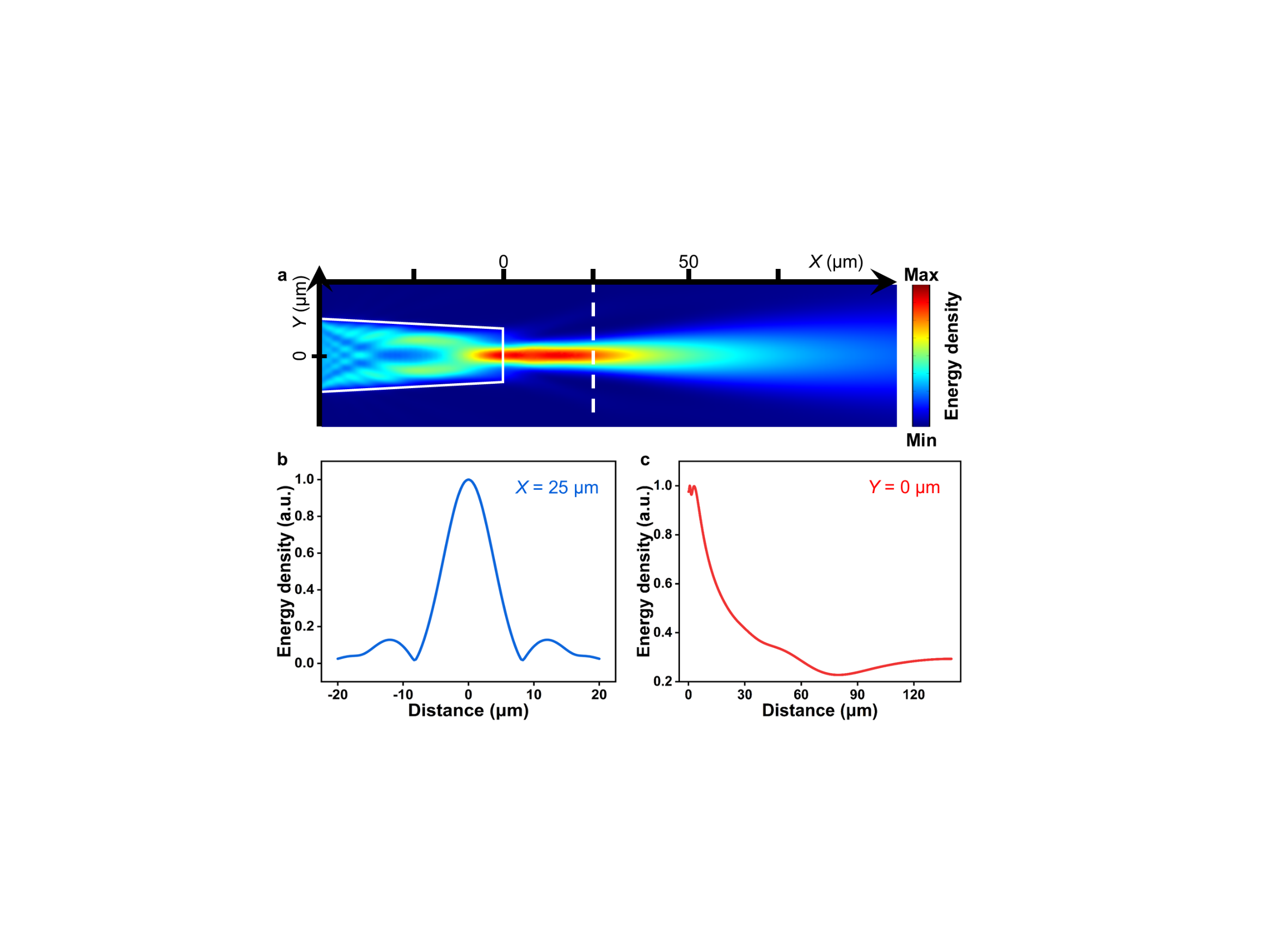


**Fig. S4.** **Energy estimation of SMOFP.** (**a**) Simulated optical energy density distribution output from the SMOFP. (**b**) Normalized energy density at the focal plane (*X* = 25 µm) with different Y locations. (**c**) Normalized energy density distribution along the fiber axis (*Y* = 0).


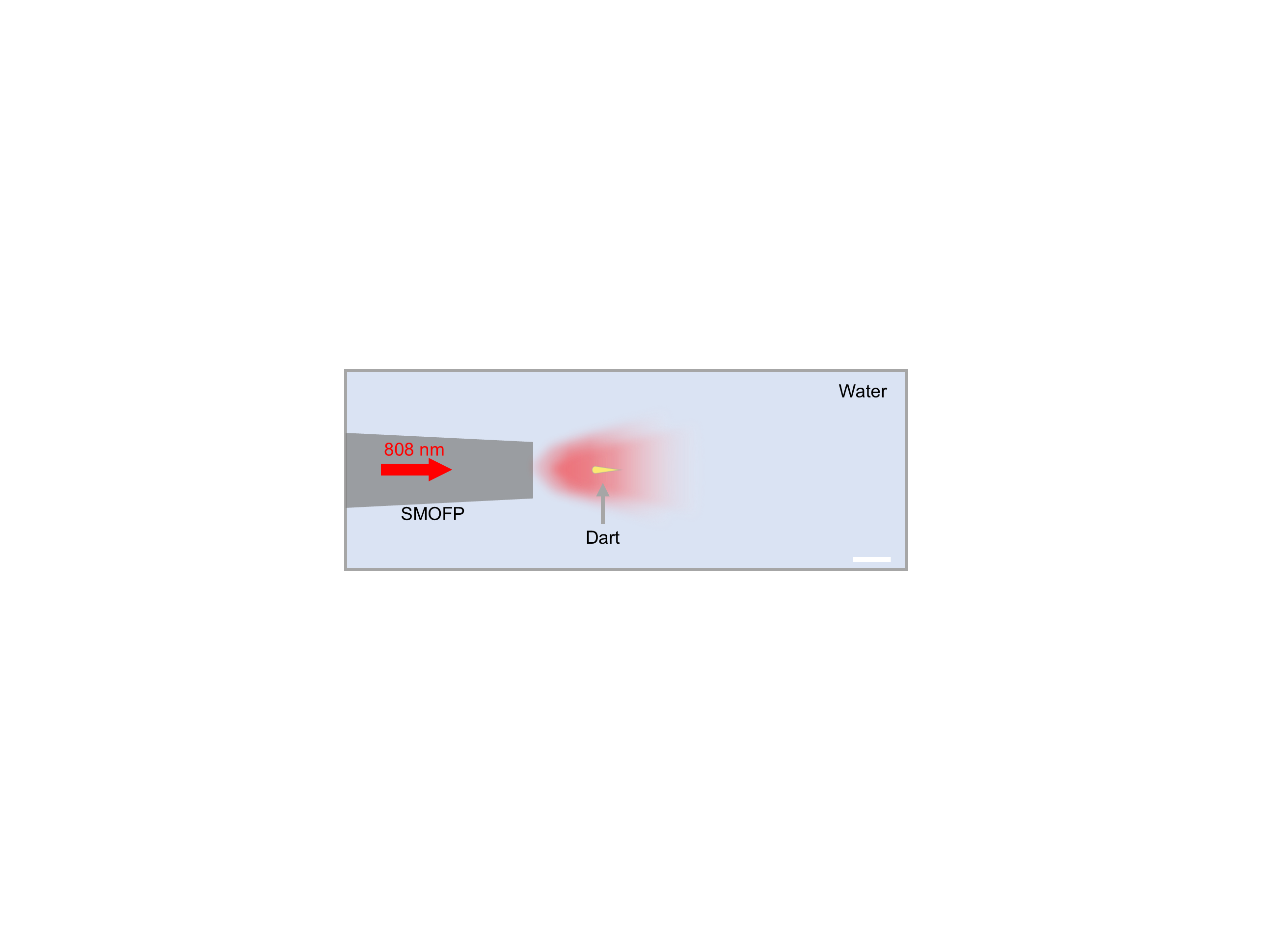


**Fig. S5.** Model of the simulation used for light field distribution and optical force calculation. Scale bars: 10 µm.

In the simulation, COMSOL Multiphysics, a finite element analysis software, was employed for computational modeling. The Electromagnetic Waves module was used for the analysis of optical field distribution surrounding a single dart under laser irradiation at 808 nm. The dart was modeled as a cone with a tip of 0.17 μm, a base of 3.1 μm, and a length of 7.5 μm, as shown in Fig. S5. The flat-end tapered SMOFP was approximated with a trapezoidal geometry, the diameter of the cross-section of the fiber end is 15 μm. The medium surrounding the dart and SMOFP was assumed to be water. The refractive indices of the SMOFP, dart, and water were set to be 1.44, 1.48, and 1.33, respectively. Initial conditions included a water temperature of 25 °C and a laser power of 150 mW. The optical force (*F*) was calculated by integrating the Maxwell stress tensor around the dart, given by *Fx = int_surf(emw.unTx)****.***

The Solid Mechanics module was used for the analysis of pressure field distribution on a cell membrane stimulated by a dart. The dart was modeled as a cone with a tip of 0.17 μm, a base of 3.1 μm, and a length of 7.5 μm. The targeted segment of the cell membrane was represented as a cylindrical section with a diameter of 2 μm and a height of 0.02 μm. An elastoplastic model was adopted to characterize the mechanical properties of the cell membrane, with the following material properties: a Young's modulus of 2.1E^5^ MPa, a Poisson's ratio of 0.3, and a density of 7850 kg m^-^³. The pressure (*P*) was calculated by integrating the stress tensor across the surface surrounding the dart, given by *P = int_surf(solid.pm)*.


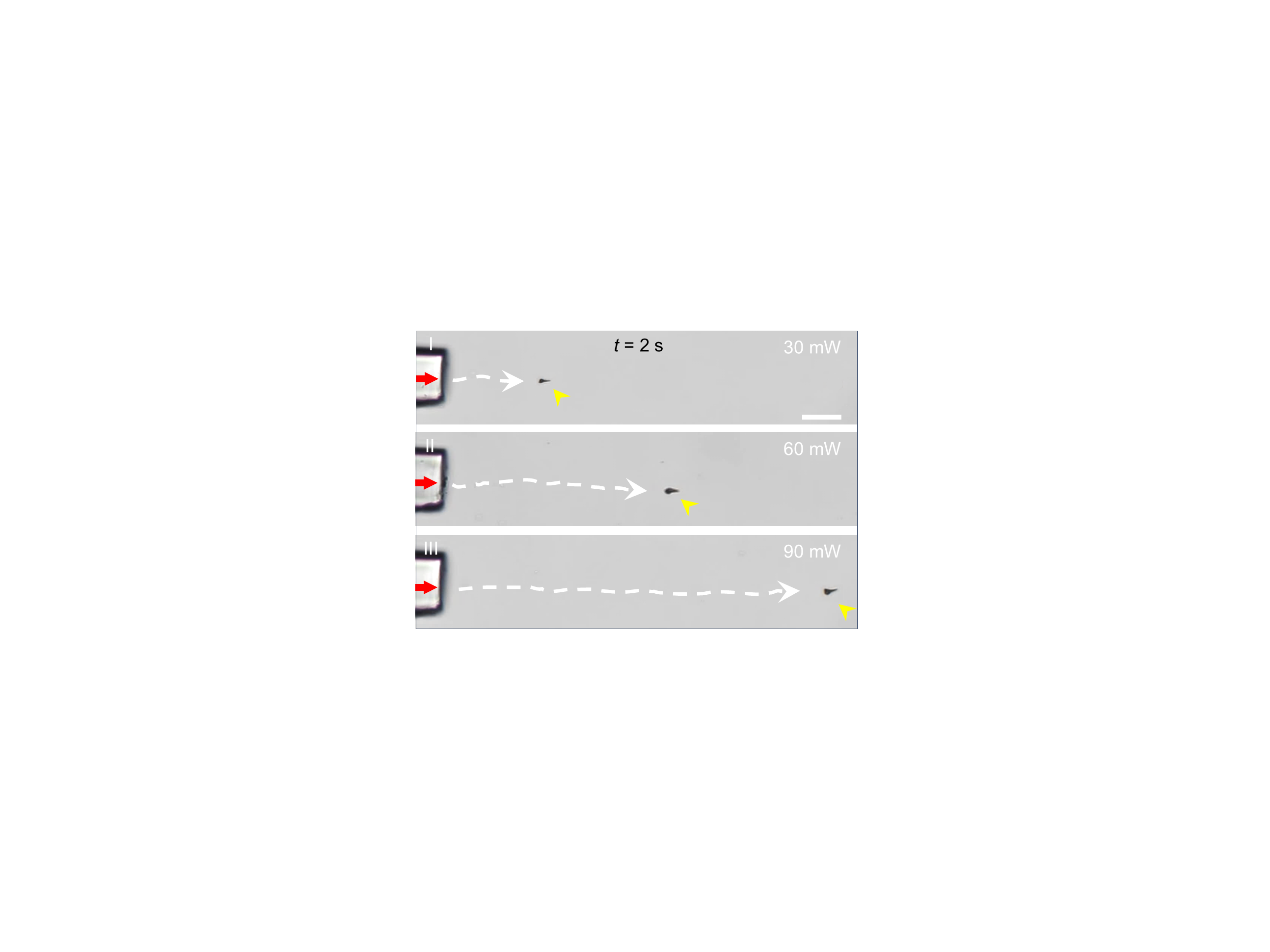


**Fig. S6.** **Movement of darts at different laser powers.** White dashed line shows the actual path of the bio-dart. The red and yellow arrows indicate 808 nm laser beam and bio-dart, respectively.


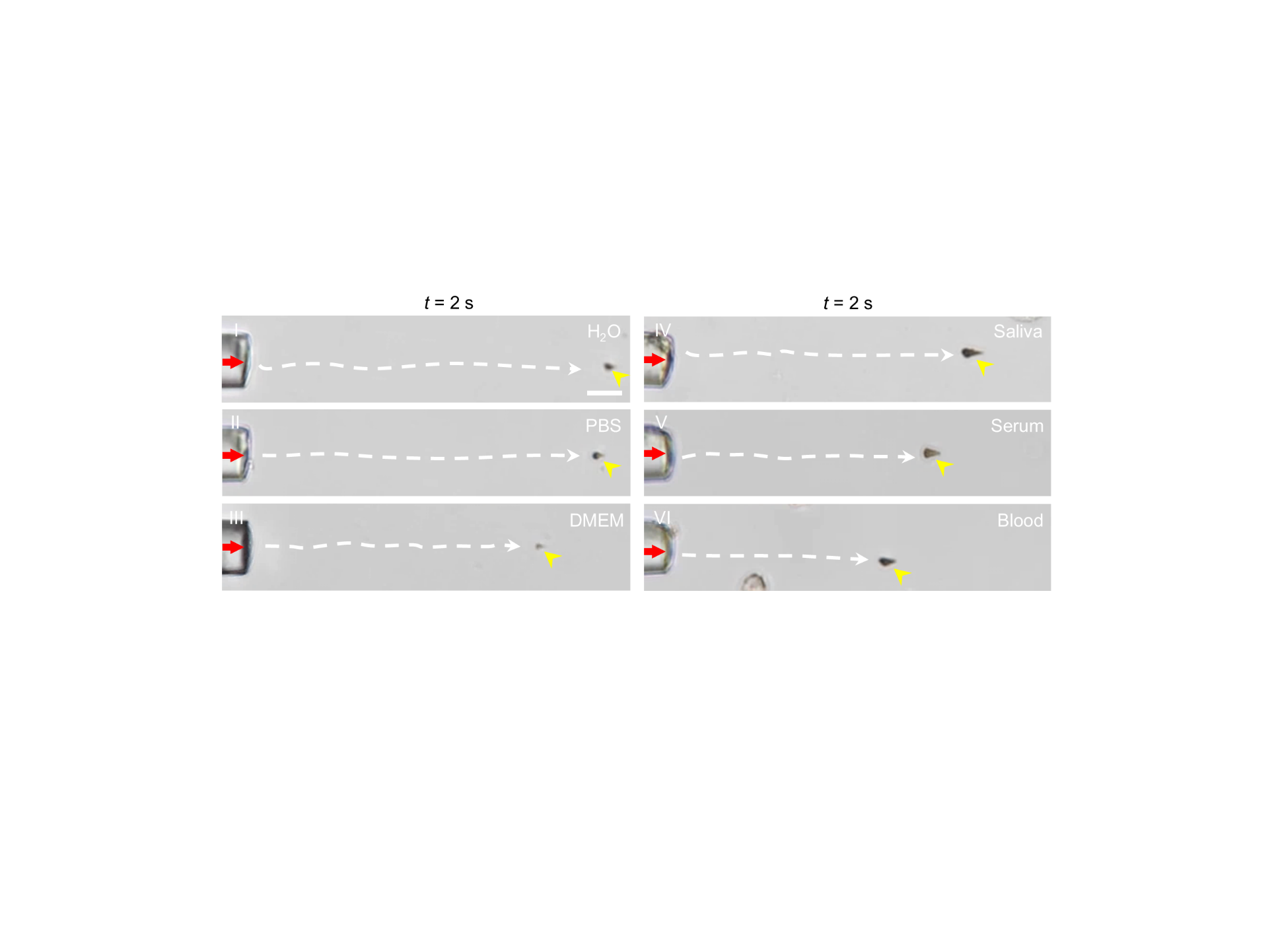


**Fig. S7. Movement of darts in various biological media.** White dashed line shows the actual path of the bio-dart. The red and yellow arrows indicate 808 nm laser beam and bio-dart, respectively.


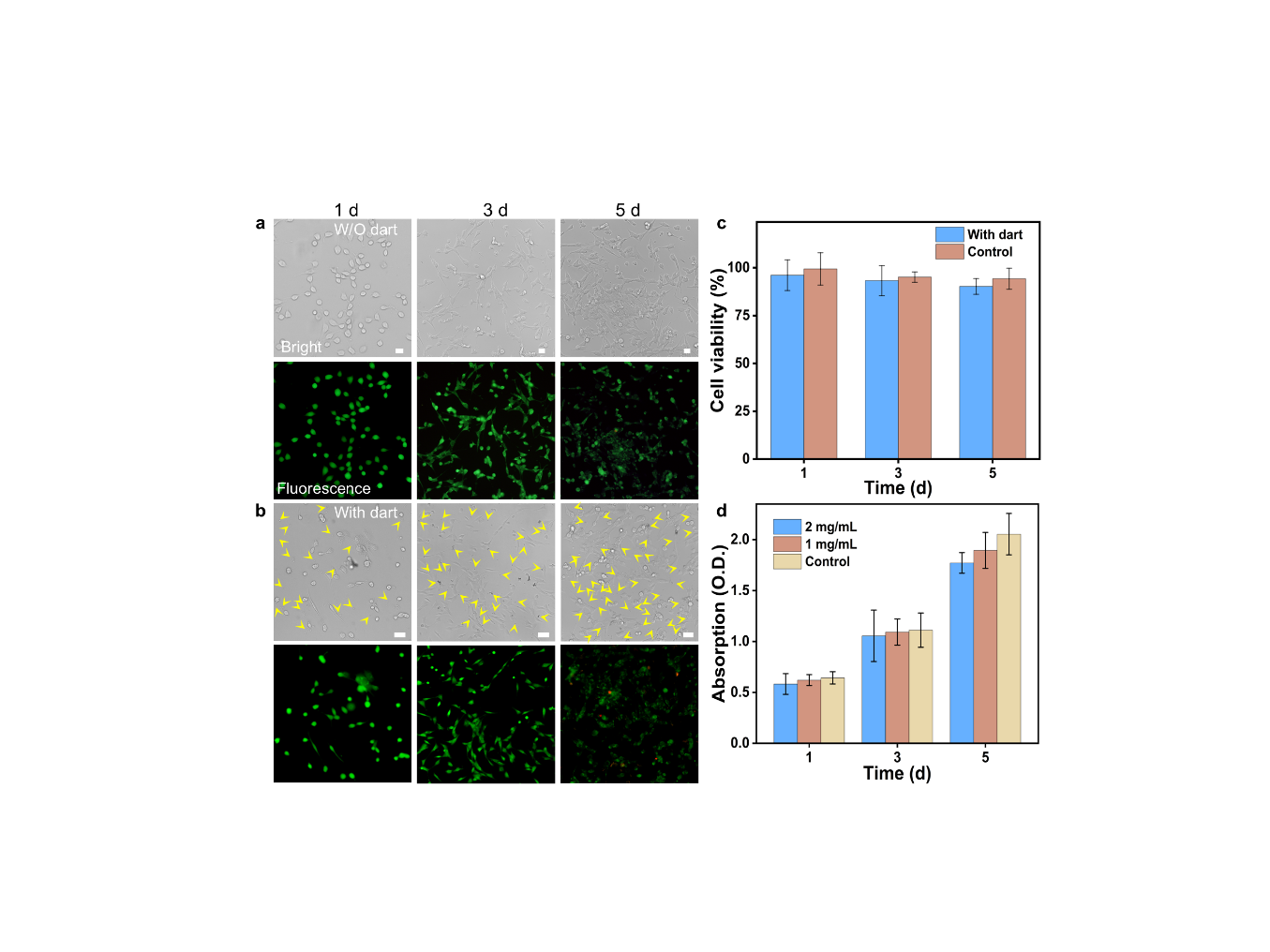


**Fig. S8.** **Sterility and biocompatibility evaluation of bio-dart.** (a,b) Bright field image and corresponding fluorescence image showing the culturing of HT22 cells treated (a) without or (b) with darts in 5 days. The yellow arrows indicate the position of dart, green fluorescence for living cells. Scale bar: 20 μm. (c) Cell viability after co-culturing with darts. (d) Absorption density of cultured cells with cell counting Kit-8 assays (CCK-8) at 450-nm wavelength, showing the proliferation ability of the neurons after co-culturing with darts with 5 days.

In the experiments, we used dual-fluorescent calcein-AM/propidium iodide (PI) assay to test the viability of the cell. The calcein-AM assay is based on the conversion of the cell permeant nonfluorescent calcein-AM dye to the fluorescent calcein dye (green fluorescence) by intracellular esterase activity in live cells. PI labels dead effector cells, as well as dead target cells once their overall plasma membrane are compromised. In the experiments, we tested the cell viability after cells co-cultured with darts at different time. As shown in Fig. S8a and S8b, after 5-days incubation of calcein-AM/PI assay, all the cells were fluorescent green, indicating the cells were still alive. The dart (5 d) exhibits negligible toxicity. The cell viability was > 85% (Fig. S8c). In addition, we also evaluated the proliferation ability of the neurons after co-culturing with darts. As shown in Fig. S8d, we find that compared with neurons cultured in normal state, no obvious influence on the proliferation was observed with the concentration of darts up to 2 mg mL^-1^. The above experimental results demonstrate that dart have good biocompatibility with limited toxicity to HT22 cell, and can be applied safely in biological environments.


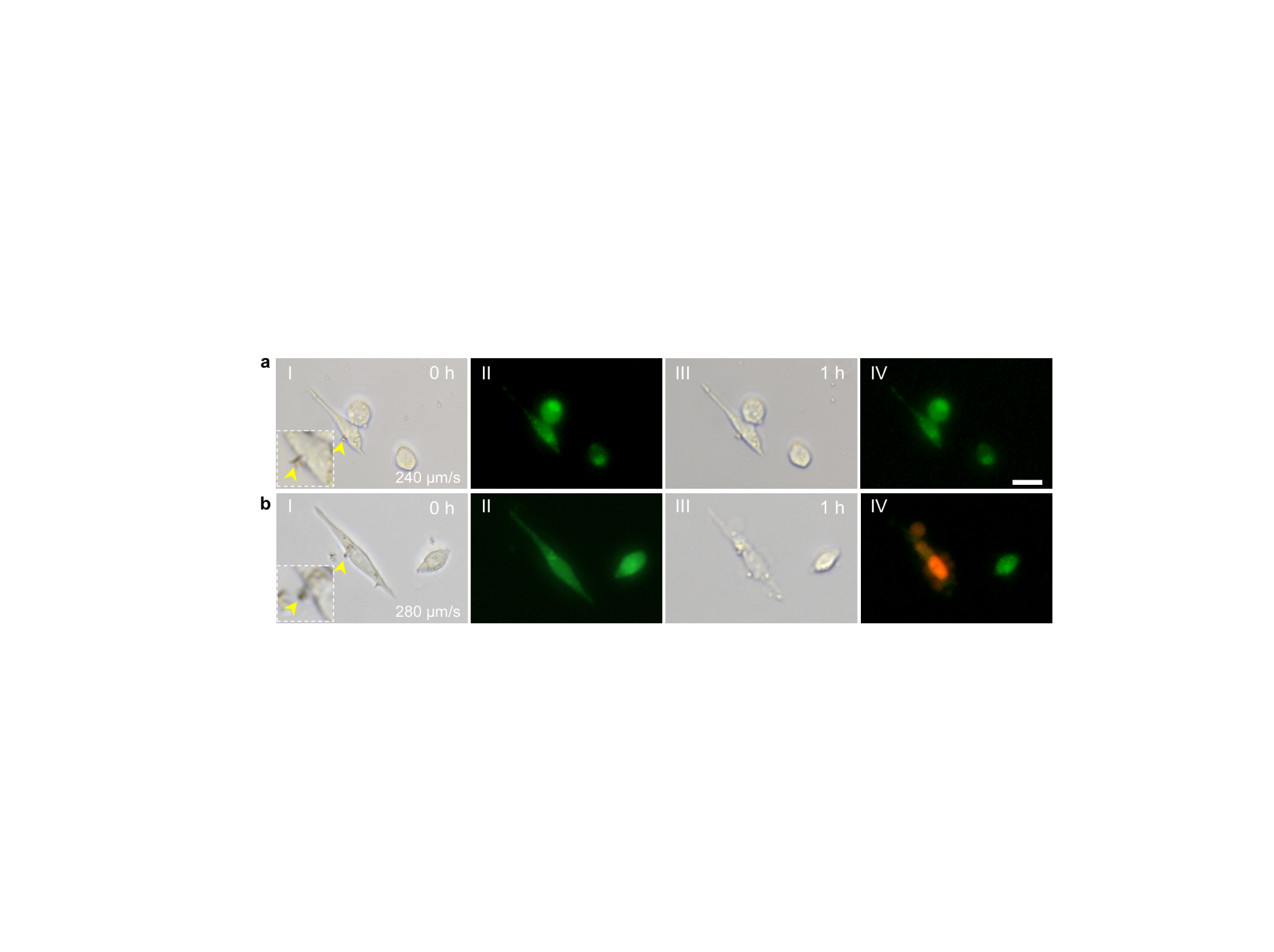


**Fig. S9.** **Effect of bio-dart shooting speed on cell viability.** (a) Bio-dart shooting toward cell membrane at speed of 240 μm s^-1^. The cell membrane was pierced by the dart, but the viability was not affectd. (b) Bio-dart shooting toward cell membrane at speed of 280 μm s^-1^. The cell membrane was pierced by the dart, and the viability was affected. Scale bar: 20 μm.


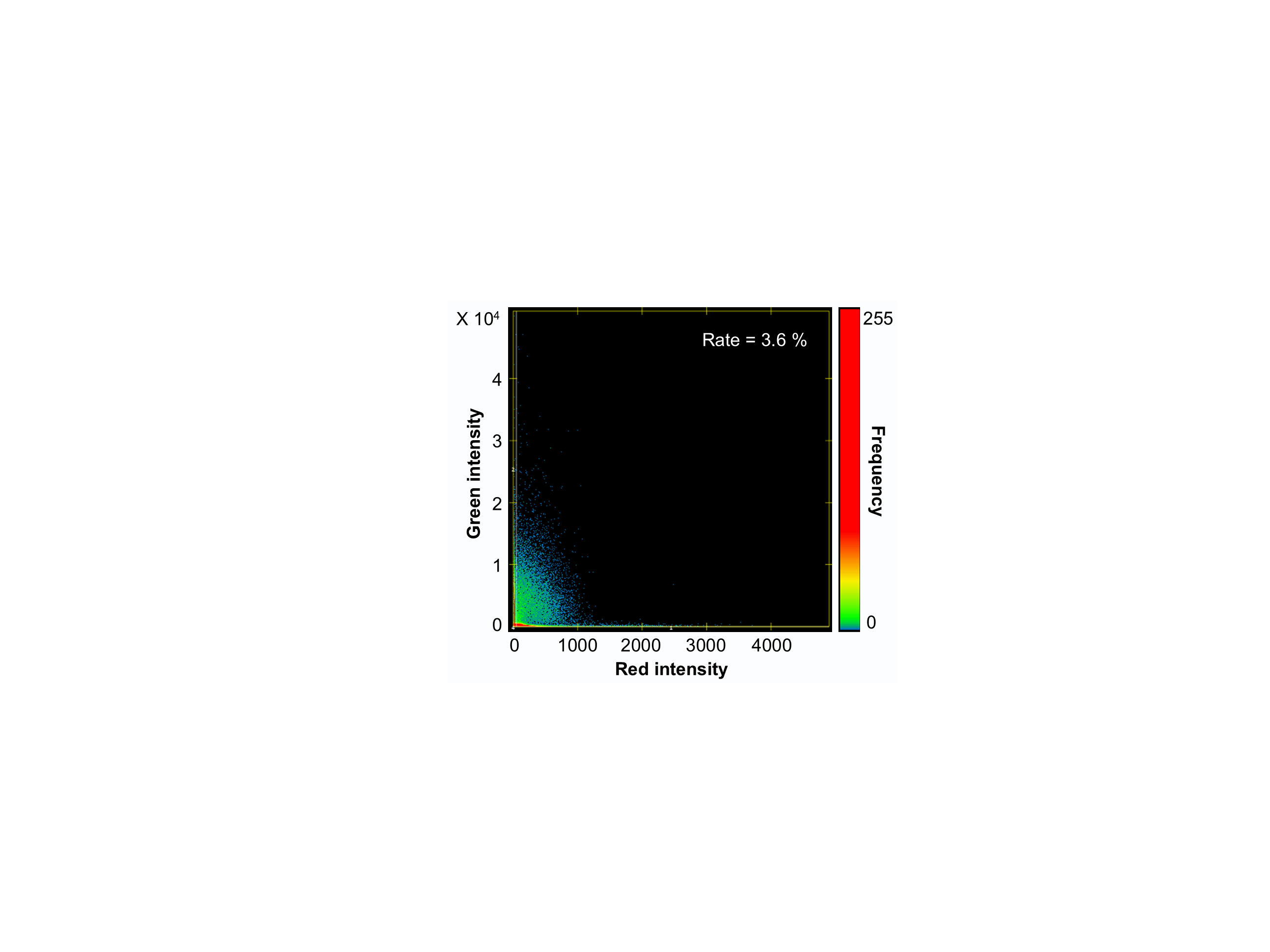


**Fig. S10. Contact between dart and cell.** Immunofluorescence co-localization scatter plot of the grayscale intensity extracted from Figure 2k. The result shows that the tip section of the dart entered the cell, and have a 3.6% junction area between the dart and the cell. The 3.6% overlap was concluded from the colocalization of the red fluorescence of dart and the green fluorescence of neuron cell, which was obtained using the plug-in (Coloc 2) in the software ImageJ. This image was captured after 10 s of the bio-dart hitting the neuronal cell.


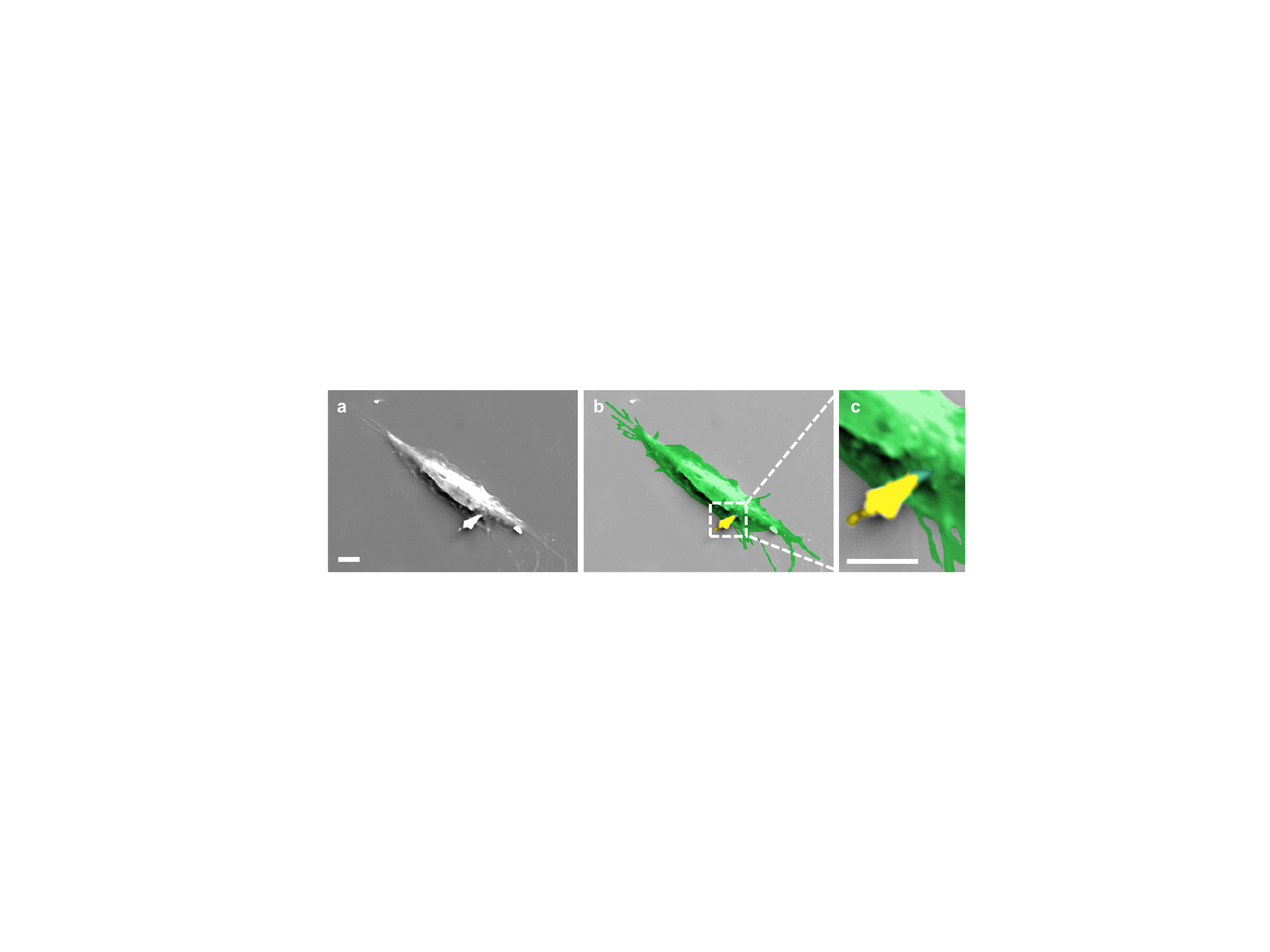


**Fig. S11. SEM image showing the tip of a dart embedded in cell membrane after shooting.** (a) The captured SEM image, (b) False-color image showing the dart (yellow) embedded in the neural cell (green). (c) Enlarged view showing the dart tip embedded in the cell membrane. The dark green indicates the overlapped region of the dart tip and cell membrane after embedding. Scale bar: 5 μm.


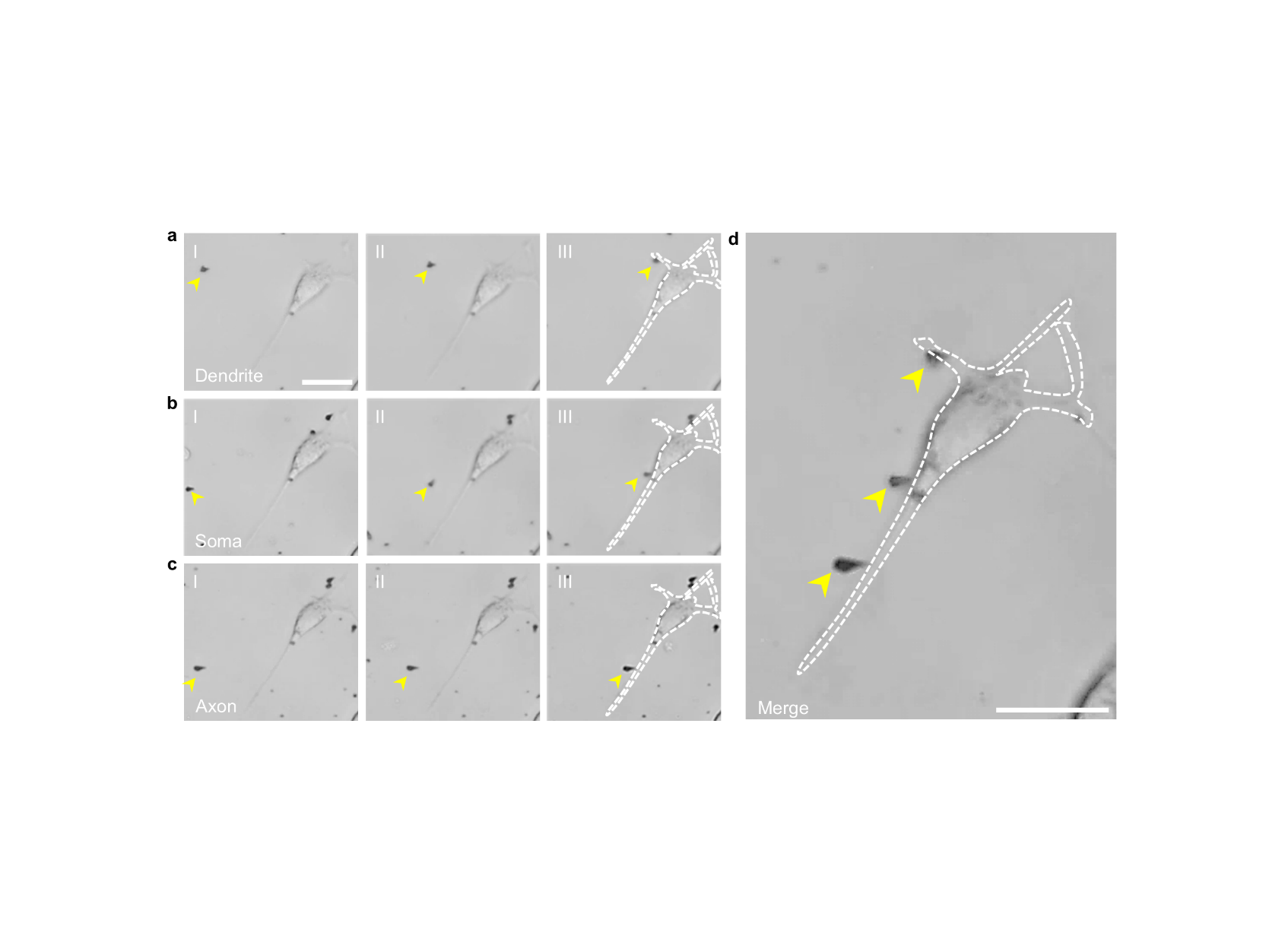


**Fig. S12. Precise targeting of darts on subcellular level.** (**a-c**) Time-lapse images illustrating that darts target dendrite(**a**), soma (**b**), and axon (**c**) of cell, respectively. (**d**) Merged images illustrating the end-on attachment of three small opening-oriented darts at small opening onto the same neural cell. White dashed structures indicate the position of cell, Red dashed lines indicate the targeted area and yellow arrows indicate the position of dart. Scale bar: 20 μm.


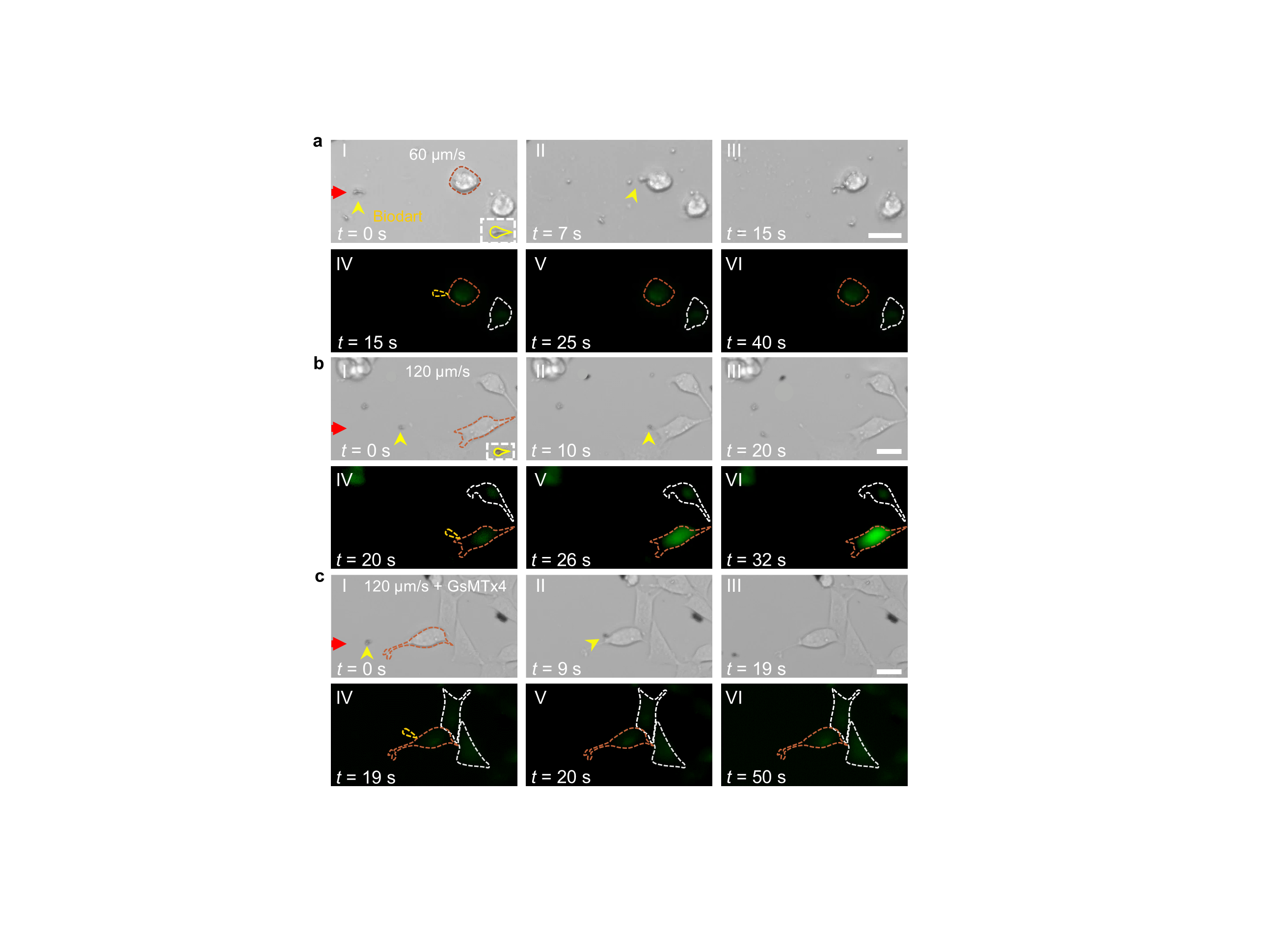


**Fig. S13.** **Targeted activation of Piezo1 channel.** Representative time-lapse microscopy and fluorescence imaging of HT22 cell stimulated by dart as 60 μm s^-1^ (**a**) 120 μm s^-1^ (**b**) 120 μm s^-1^ + GsMTx4 **(c**) White and brown dashed structures indicate the position of cell, red arrows indicate direction of the laser and yellow arrows indicate the dart. Scale bar: 20 μm.

Piezo1 channel is a mechanosensitive cation channel, which, upon mechanical stimulation, opens the channel proteins on the cell membrane, allowing extracellular cations such as K^+^, Na^+^, Ca^2+^ to enter the cell. Ca^2+^ signaling contributes to the growth and differentiation of neuronal cell. And transient change of Ca^2+^ regulate neuronal differentiation, axon growth, and radial glial cell proliferation. We steered dart into cell culture medium to stimulate HT22 cell. Under the guidance of optical scattering force, darts approach the target HT22 cells. The changes in intracellular calcium were monitored in real time. After the darts reached the vicinity of the target cells (marked by red arrows), the Ca^2+^ concentration transients in the HT22 cells were monitored by the Ca^2+^-responsive fluorescent probe Fluo-4 AM. As shown in Fig. S13b, the intensity of intracellular Ca^2+^ fluorescence gradually increasing with hitting velocity of 120 μm s^-1^. But, no significant change in Ca^2+^ fluorescence intensity was observed for mechanical stimulation of darts with hitting velocity of 60 μm s^-1^ and pre-treated with GsMTx4 (Fig. S13a, c).


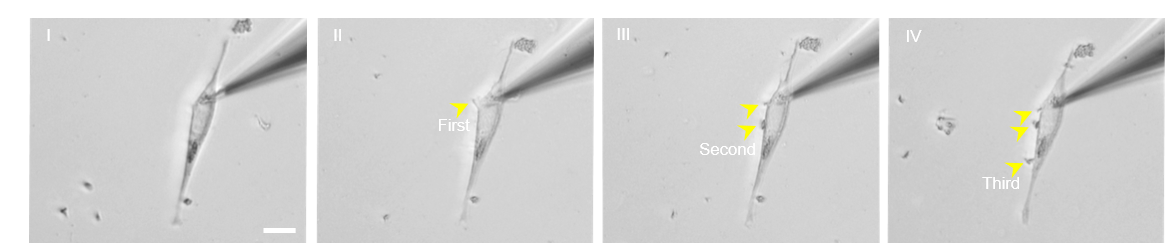


**Fig. S14.** Microscopic images showing the repeated stimulation of the same neuron with three darts (yellow arrow indicated). Scale bar: 20 μm.


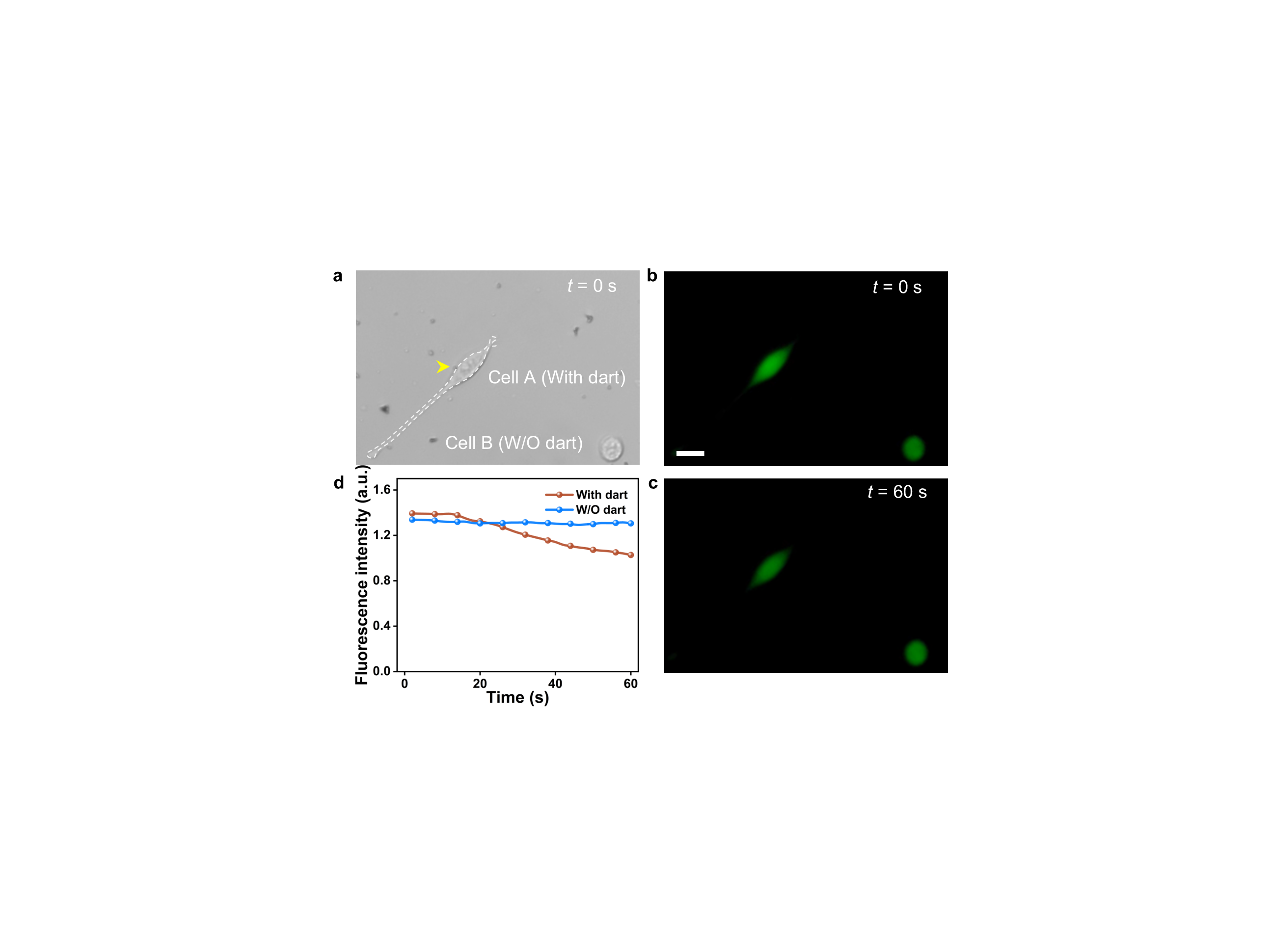


**Fig. S15. Neural silencing via dart.** (a) Bright-field microscopic image showing GsMTx4-loaded dart (yellow arrow indicated) shooting onto a target cell. (b, c) Fluorescent images showing Ca^2+^ response after dart shooting. (d) Fluorescence intensity of Ca^2+^ signal as a function of time after stimulation. Scale bar: 20 μm.

**
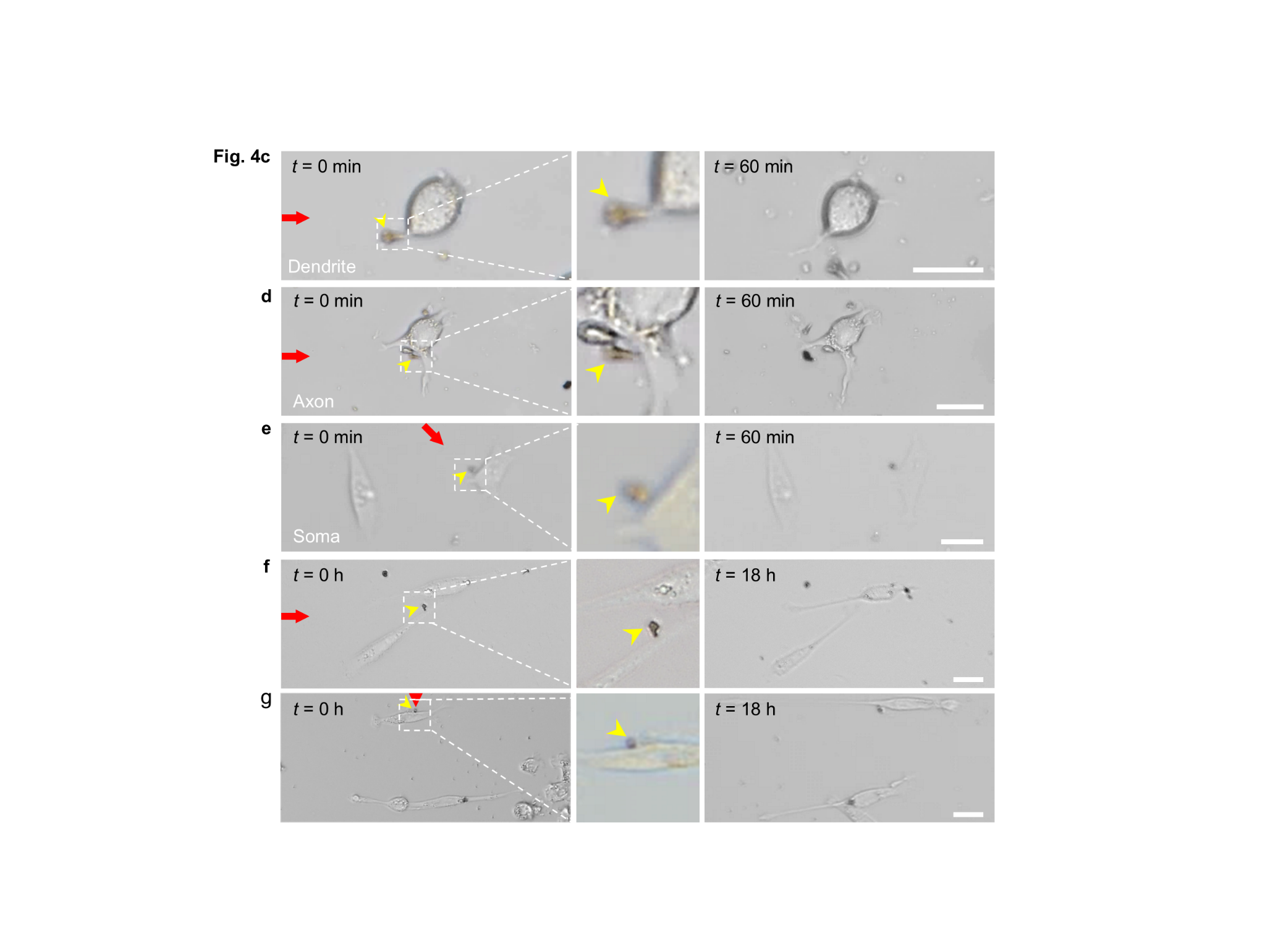
**

**Fig. S16.** The original figures for Fig. 4c-g without the dashed curve overlaying cell boundary. The yellow arrows indicate dart. Scale bar: 20 μm.


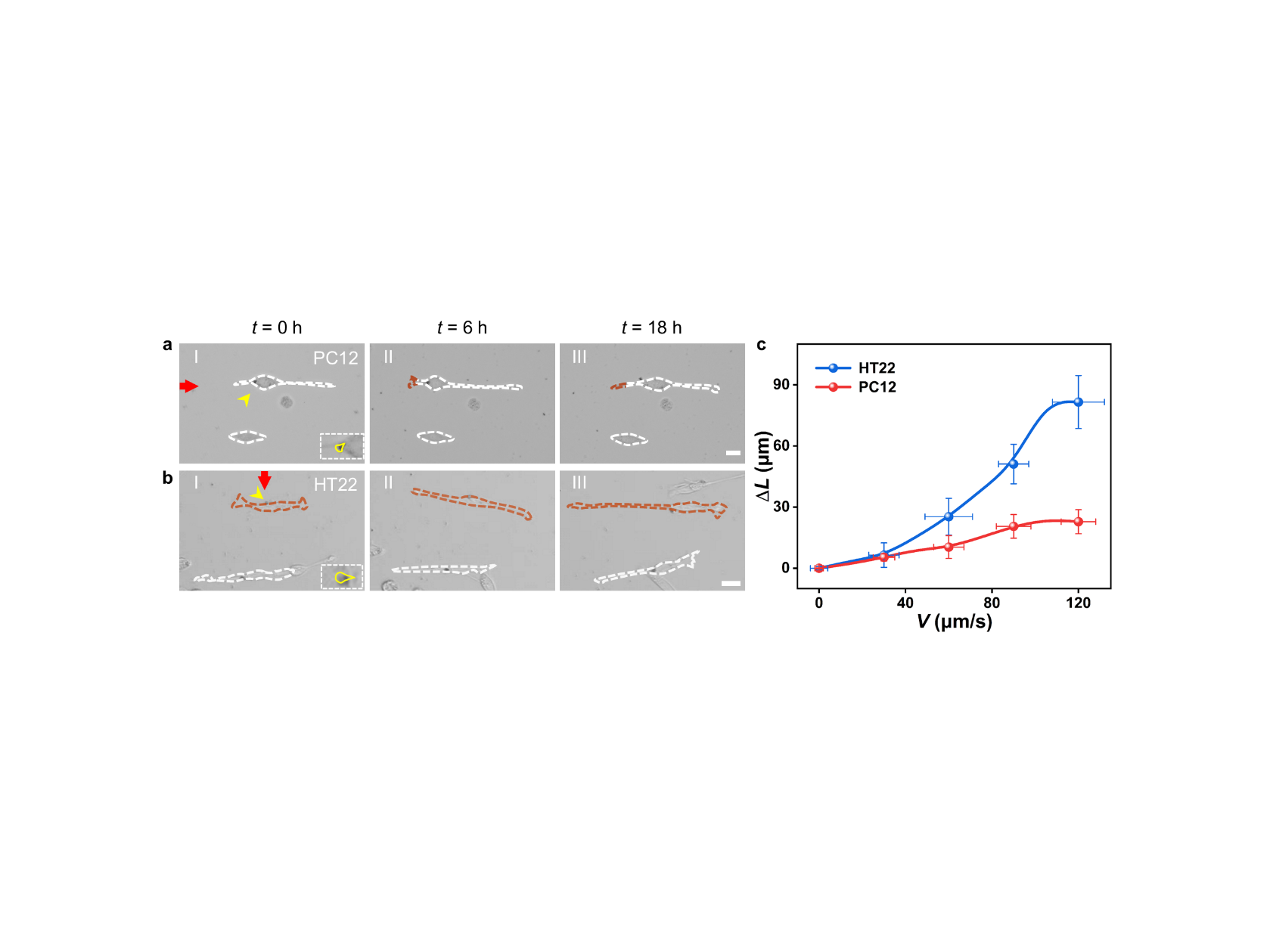


**Fig. S17. The applicability of darts for nenronal modulation.** Representative time-lapse images showing the growth of cell, (**a**) PC12 and (**b**) HT22. (**c**) Growth length of cell (HT22, PC12) as a function of the different velocities. White dashed structures indicate the position of cell, brown dashed indicate growth direction of cell, red arrows indicate direction of the laser and yellow arrows indicate the dart. Scale bar: 20 μm.


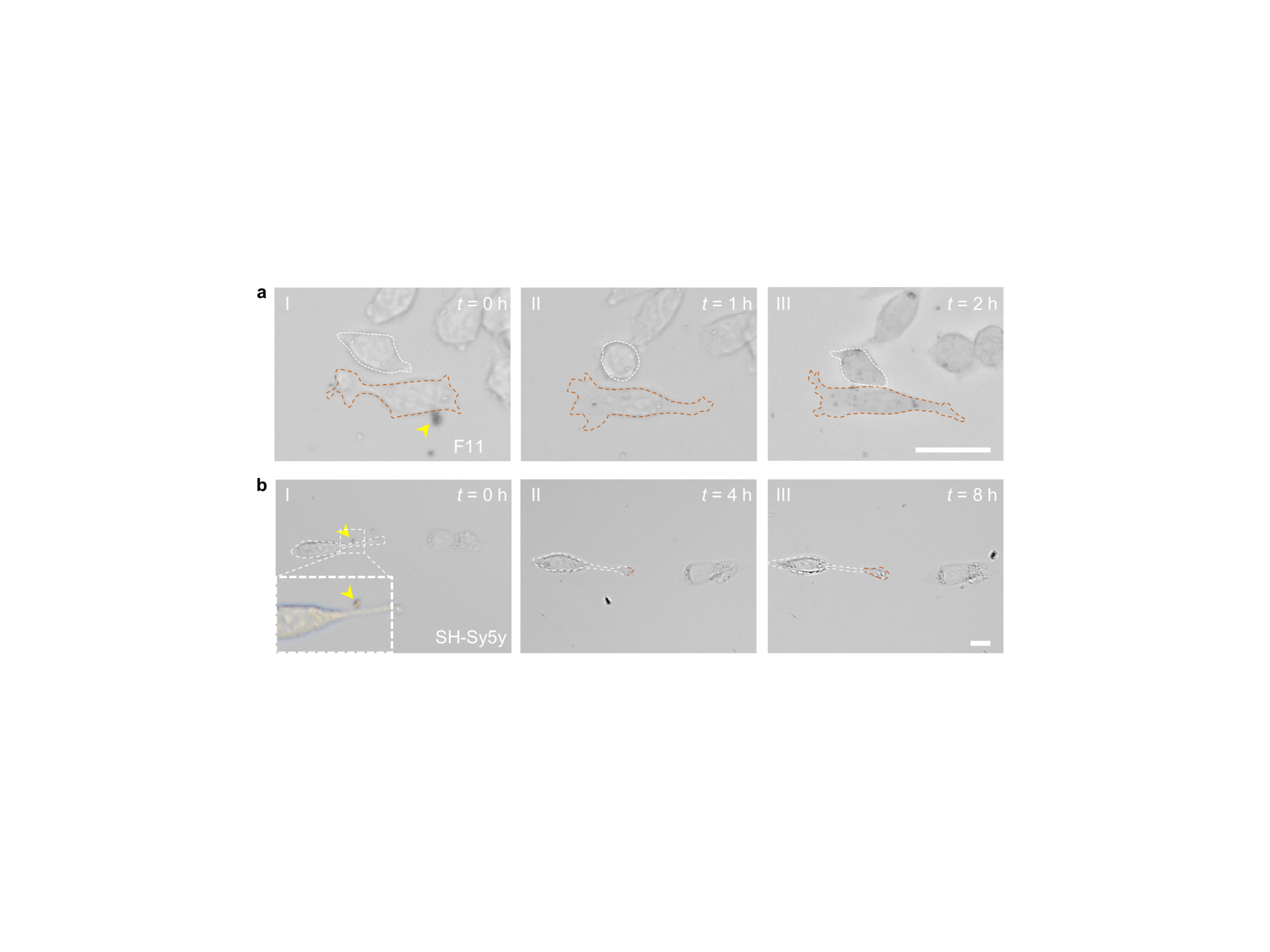


**Fig. S18.** Microscopic images showing precision modulation of different neural cells of (a) F11, (b) SH-Sy5y. Inset in (b) shows an enlarged view of the stimulated area. Red curves show the boundary of target cell with stimulation, while white curve shows the neighboring cell without stimulation. Scale bar: 20 μm.


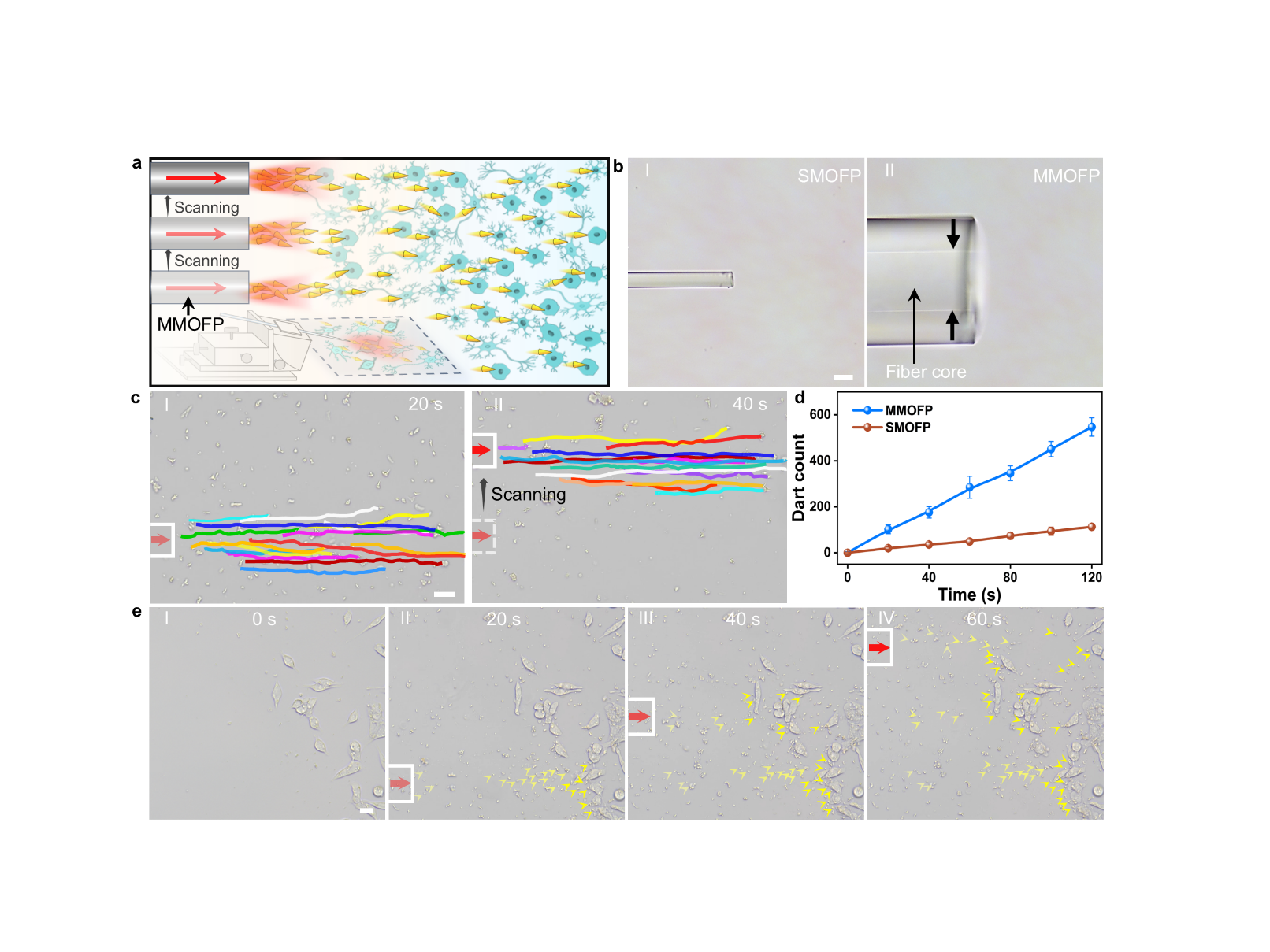


**Fig. S19.** **High-throughput dart shooting.** (a) Schematic illustration of high-throughput dart shooting for large-scale neuronal stimulation via moving/scanning of a MMOFP. (b) Microscopic images showing a tapered flat-end SMOFP and flat MMOFP. (c) Microscopic images showing high-throughput shooting of darts by MMOFP. Different colored curves show the trajectories of multiple darts moving. (d) Darts count of shooting by SMOFP and MMOFP as a function of time. (e) Microscopic images showing high-throughput shooting of darts toward large-scale neuronal cells. White curve indicates the position of MMOFP for dart shooting, yellow arrows indicate darts shooting toward neuronal cells. Scale bars: 20 µm.


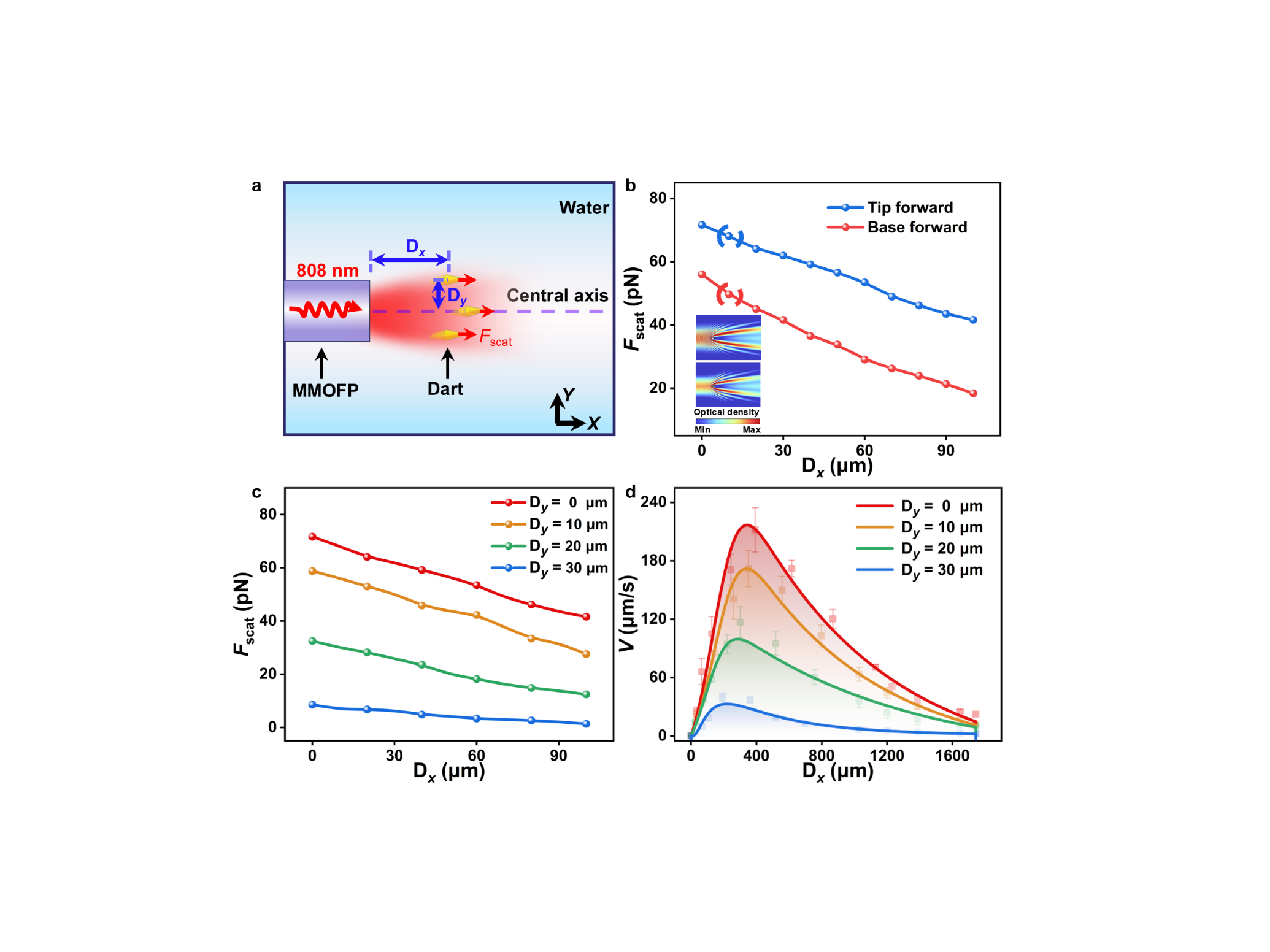


**Fig. S20.** (**a**) Schematic illustration of bio-dart shooting using MMOFP. (**b**) Calculated optical scattering force (*F*_scat_) of bio-dart (both tip forward and base forward) on the central axis of the MMOFP as a function of distance from the fiber tip, inset shows the simulated optical intensity fields of darts with two different orientations (upper: tip forward, lower: base forward). (**c**) Calculated optical scattering force (*F*_scat_) exerted on darts with tip forward at different positions from the central axis of the MMOFP as a function of distance to the MMOFP end. (**d**) Velocity of darts (tip forward) at different positions from the central axis of the MMOFP as a function of distance to the MMOFP end at power of 200 mW.


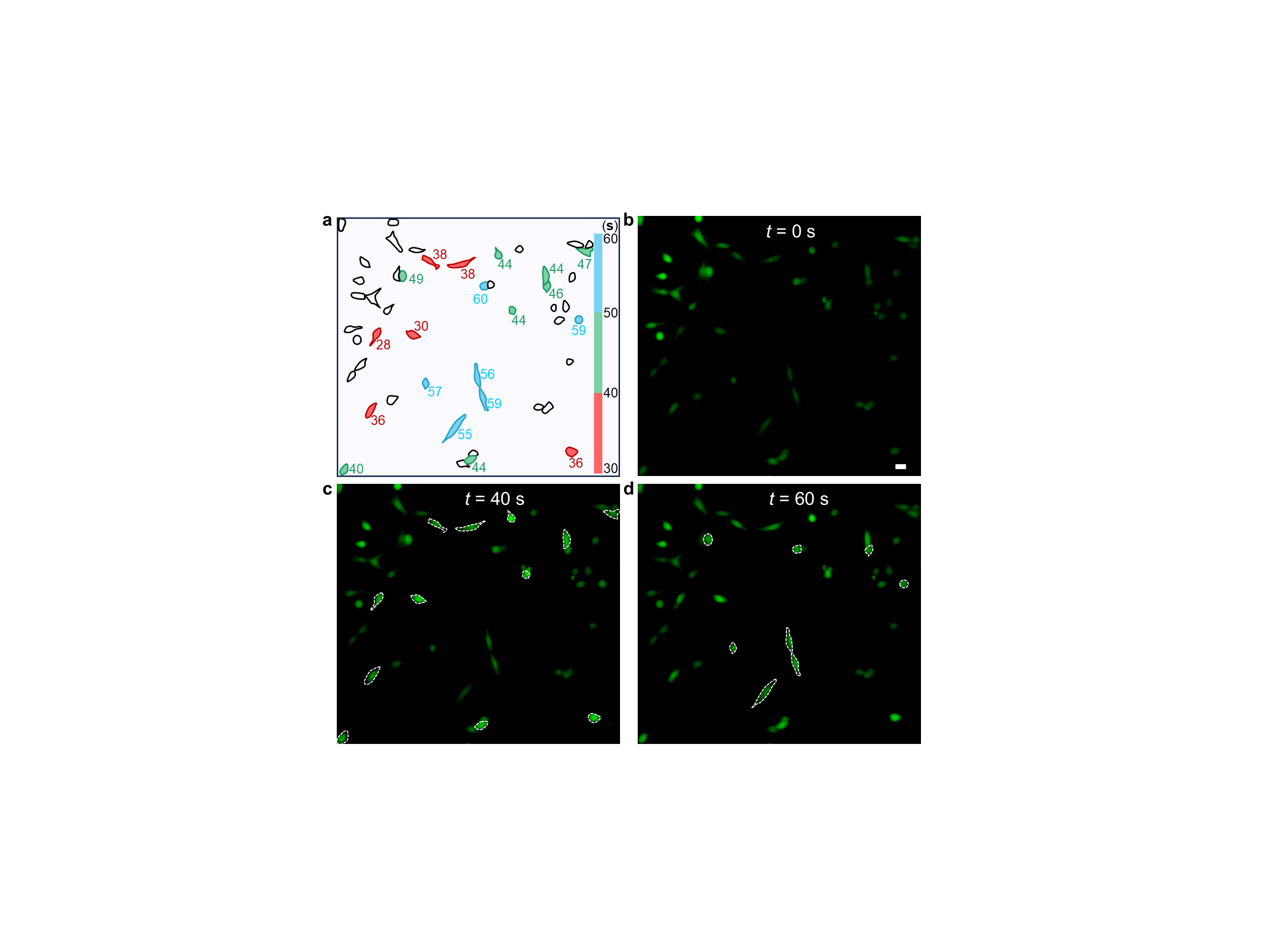


**Fig. S21.** **Large-scale stimulation of HT22 cells after dart shooting.** (a) Contour maps showing the spatial location of the cells. Different colors show the cells activated at different times as indicated by the color bar. Cells with shaded color are the activated cells, while black are the inactivated cells. (b-d) Time-lapse image sequence of the location of the activated neuronal cells. White curves show the boundary of target cell with stimulation. Scale bars: 20 µm.


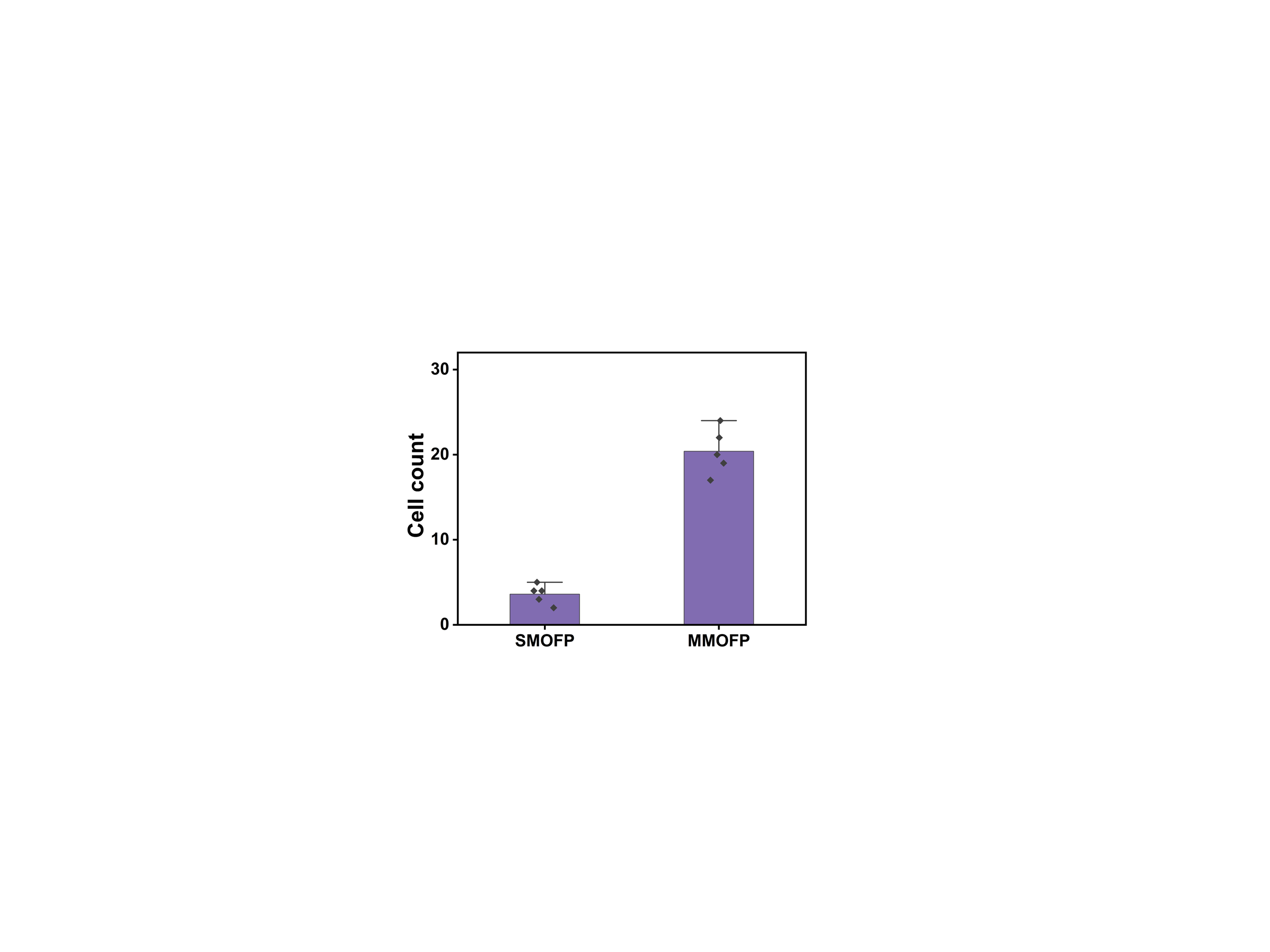


**Fig. S22.** Number of neurons excited within a minute using SMOFP and MMOFP.

**
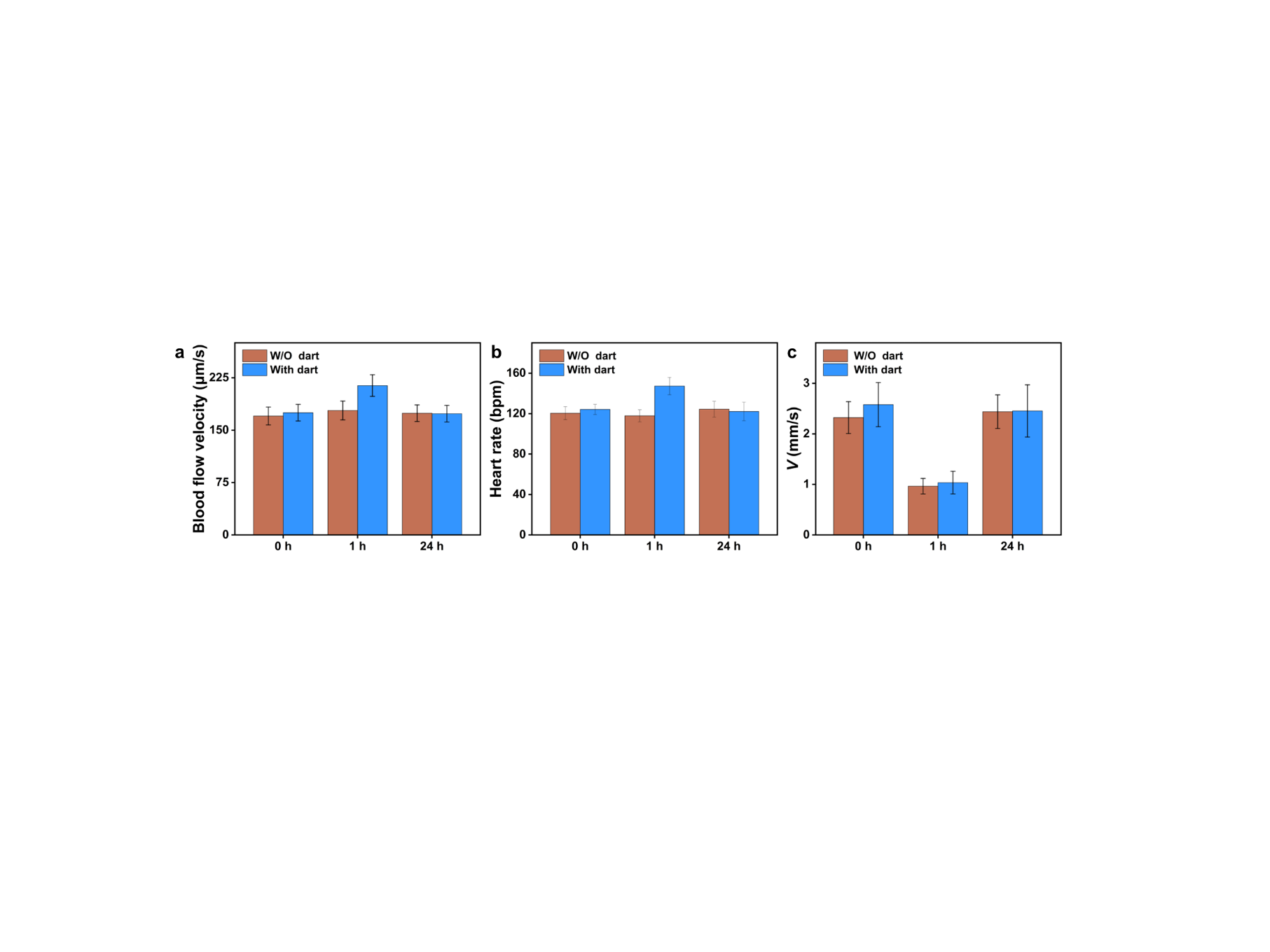
**

**Fig. S23.** Comparison of zebrafish (a) blood flow velocity, (b) heartbeat, and (c) moving velocity with and without dart shooting.

**Description of Supporting videos**

Video S1. Orientation and acceleration of Bio-dart.

Video S2. Bio-dart shooting in different bio-microenvironments.

Video S3. Bio-dart shooting towards different targets.

Video S4. Bio-dart shooting toward a single neuronal cell.
